# Optimal@NRW: optimized acute care of nursing home residents using an intersectoral telemedical cooperation network — study protocol for a stepped-wedge trial

**DOI:** 10.1186/s13063-022-06613-1

**Published:** 2022-09-27

**Authors:** David Brücken, Jenny Unterkofler, Sophie Pauge, Jonas Bienzeisler, Christian Hübel, Sebastian Zechbauer, Rolf Rossaint, Wolfgang Greiner, Birthe Aufenberg, Rainer Röhrig, Leo Cornelius Bollheimer, Optimal@NRW Research Group, Julia Stingl, Martina Ziefle, Julia Offermann, Pia Erdmann, Albrecht Eisert, Lea Koech, Saskia Wilhelmy, Judith Steinfeld, Dominik Groß, Andreas Follmann, Michael Czaplik, Johannes Pollmanns, Thomas Krafft, Simone Böbel, Maresa Neuerer, Jörg Christian Brokmann

**Affiliations:** 1 Department for Acute and Emergency Medicine, University Hospital RWTH Aachen, 52074 Aachen, Germany; 2 Department for Health Economics and Health Care Management, School of Public Health, Bielefeld University, 33615 Bielefeld, Germany; 3 Institute of Medical Informatics, RWTH Aachen University, 52074 Aachen, Germany; 4 Department of Anaesthesiology, University Hospital RWTH Aachen, 52074 Aachen, Germany; 5 Department of Geriatrics, University Hospital RWTH Aachen, 52074 Aachen, Germany

**Keywords:** Telemedicine, Geriatrics, Nursing home, Outpatient care, Hospital admission, Ambulatory care-sensitive hospitalizations, Teleconsultation, Telephysician

## Abstract

**Background:**

Increasing life expectancy is associated with a growing number of people living in nursing homes, while the availability of outpatient medical care, especially from family doctors, is stagnating in this sector. Consequently, numerous and often avoidable, low-threshold hospitalizations of nursing home residents are observed. This results in unnecessary use of resources such as emergency services and emergency rooms as well as in potential health risks to the nursing home residents related to hospitalization. This study aims to improve this healthcare gap by implementing an intersectoral telemedicine approach.

**Methods:**

Twenty-five nursing homes are participating and provided with telemedical equipment to perform teleconsultations. Additionally, an early warning system and a digital patient record system are implemented. Telephysicians based at RWTH Aachen University Hospital are ready to support the nursing homes around the clock if the family doctor or an emergency service practice is not available in time. Mobile non-physician practice assistants from the telemedicine centre can be dispatched to perform delegable medical activities. General practitioners and the medical emergency practices also have access to the telemedical infrastructure and the non-physician practice assistants.

**Discussion:**

Optimal@NRW adds a telemedicine component to standard care — combining elements of outpatient and inpatient health care as well as emergency service practices — to enable timely medical consultation for nursing home residents in case of the development of an acute medical condition. In addition to optimized medical care, the goal is to reduce unnecessary hospital admissions. The intersectoral approach allows for the appropriate use of resources to match the individually needed medical treatment.

**Trial registration:**

ClinicalTrials.govNCT04879537. Registered on May 10, 2021

**Supplementary Information:**

The online version contains supplementary material available at 10.1186/s13063-022-06613-1.

## Administrative information

Note: the numbers in curly brackets in this protocol refer to SPIRIT checklist item numbers. The order of the items has been modified to group similar items (see http://www.equator-network.org/reporting-guidelines/spirit-2013-statement-defining-standard-protocol-items-for-clinical-trials/).

**Table:** 

Title {1}	Optimal@NRW - Optimised acute care of geriatric patients using an intersectoral telemedical cooperation network - around the clock
Trial registration {2a and 2b}.	ClinicalTrials.gov Identifier: NCT04879537 (registered May 10, 2021)
Protocol version {3}	Version 02, 09.03.2021
Funding {4}	The trial is funded by the Innovation Fund of the Federal Joint Committee for the Promotion of New Forms of Care (§92a para. 1 SGB V). The provided funding period lasts from April 1, 2020 to March 31, 2024 (grant number: 01NVF19015).
Author details {5a}	^1^Department for Acute and Emergency Medicine, University Hospital RWTH Aachen, Aachen, Germany ^**2**^Department for Health Economics and Health Care Management, School of Public Health, Bielefeld University, Bielefeld, Germany ^3^Institute of Medical Informatics, RWTH Aachen University, Aachen, Germany ^4^Department of Anaesthesiology, University Hospital RWTH Aachen, Aachen, Germany ^4^Department of Geriatrics, University Hospital RWTH Aachen, Aachen, Germany
Name and contact information for the trial sponsor {5b}	DLR (Department Health) Michael Kalicinski Heinrich-Konen-Str. 1 53227 Bonn michael.kalicinski@dlr.de
Role of sponsor {5c}	The sponsor is not involved in study design; collection, management, analysis, and interpretation of data; writing of the report; and the decision to submit the report for publication.

## Introduction

### Background and rationale {6a}

Demographic change because of a continuously increasing life expectancy leads to a changing age structure, especially in Europe [1, 2]. This results in multiple challenges such as providing adequate nursing and medical care for the elderly and frequently multimorbid patients in nursing homes. Timely medical care of nursing home residents represents one of the most pressing problems because a declining number of general practitioners (GPs) is faced with an ever-increasing workload. Thus, visits of GPs to attend to acute medical conditions are often not feasible, especially in rural regions [3]. Another option is the medical on-call service that can be utilized outside of GP consultation hours, but this is often associated with long waiting times. In sum, on many occasions, prompt medical care cannot be offered because on-site care is not possible in terms of time, personnel and logistics, which leads to low-threshold hospital admissions as well as over-utilization of ambulance service and emergency physician deployments. On average, out of 100 nursing home residents, about 20 are currently admitted to the hospital once a year. In addition, out of these 20 admissions, approximately 40% are avoidable, because many of these medical deteriorations are not caused by an acute or even life-threatening illness, but reflect a gradual worsening of a pre-existing underlying condition (e.g. frailty) through, for example, urinary tract infections, pneumonia or dehydration. These cases are referred to as *ambulatory care-sensitive conditions* (ACSH) [4–6].

These conditions generate, amongst others, two major problems: first, the physicians who treat those patients in an acute setting are not familiar with the complex individual medical history, which can cause a severe discrepancy between treatment decisions and the (presumed) patient’s will. Second, hospital admissions place the patient in an unfamiliar environment and are associated with a higher complication rate for geriatric patients (e.g. development of delirium). This in turn results in higher treatment costs and an elevated individual risk of further deterioration of the patient’s medical condition. Based on all these aspects, the German Expert Council on Health Care (*Sachverständigenrat im Gesundheitswesen*, *SVR*) recommended the realignment of intersectoral provision of emergency care — including provision of outpatient emergency care, inpatient emergency care and emergency services [3, 7].

Optimal@NRW aims to re-structure acute medical care of nursing home residents by implementing a telemedicine structure that provides an additional option of acute care if the GP is not available and further functions as a new link between the previous stakeholders of emergency services, medical on-call service, GPs and emergency departments.

### Aims and objectives {7}

Firstly, we hypothesize that the implementation of a telemedicine approach for nursing home residents is associated with a decrease of the length of stay in the hospital per year and an overall improvement of outpatient medical service.

Our second hypothesis is that the telemedicine approach is associated with less frequent utilization of emergency services and improvement of intersectoral treatment of nursing home residents. Currently established standard care and the telemedicine approach are compared by predefined primary and secondary outcomes. Therefore, 25 participating nursing homes are technically equipped and coordinated by a newly organized telemedicine centre that provides 24/7/365 support by trained telephysicians and non-physician practice assistants.

### Trial design {8}

**Fig. 1:**
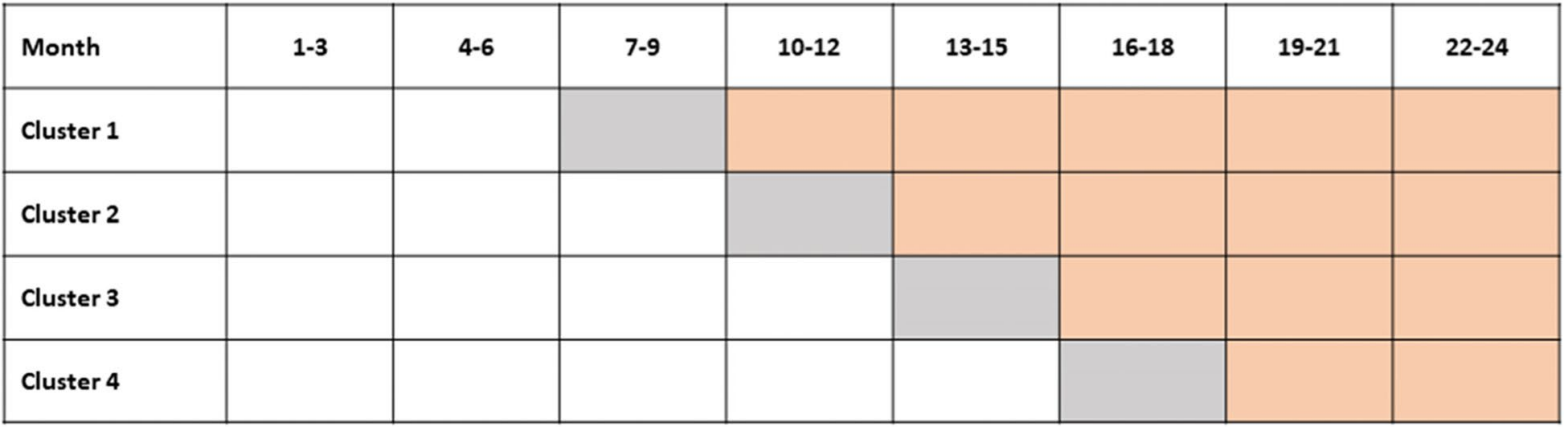
The stepped-wedge design

In order to test the aforementioned hypotheses, a multicentre, prospective, open-cohort, superiority, cluster-randomized controlled intervention trial in stepped-wedge design will be conducted. The current standard outpatient care of nursing home residents serves as control while the utilization of the telemedicine centre with all its capabilities is referred to as intervention. The trial design utilizes a stepwise switch of sequences (clusters) from control to intervention condition, where the order of crossing is randomized in terms of timing (see Fig. 1) [8]. Observation of all sequences begins simultaneously with all 25 nursing homes initially in the control phase (white). A transition phase between control and intervention is incorporated to carry out training measures for the nursing staff as well as technical installations (grey). Data collected during the transition phase are not included in the evaluation. The respective steps cover an interval of 3 months each, where clusters have to be enrolled at least for two intervals (6 months) in the control (white) and intervention (orange) cohorts in order to obtain sufficient data. The total study duration is set for 2 years, resulting in a total of eight steps. The study will be performed in 25 nursing homes, equally allocated to four sequences in order to ensure intra-cluster validity. Using an open-cohort sampling, which consists of both longitudinal and cross-sectional data, it is possible to counteract an expected high rate of mortality of nursing home residents [9].

## Methods: participants, interventions and outcomes

### Study setting {9}

The study centre including the telemedicine centre is located at the Department for Acute and Emergency Medicine at the University Hospital RWTH Aachen, Germany. All teleconsultations for the participating nursing homes will be performed there by specially trained telephysicians with a focus on geriatric and general medicine, who are available 24/7/365. The treatment options are extended by the non-physician practice assistants (*Nicht-ärztliche Praxisassistenz mit Zusatzaufgaben*), who are allowed to perform delegable medical activities on-site at the nursing homes. They are also continuously available and based at the telemedicine centre. They are dispatched to the participating care facilities for activities that can be delegated by every physician (e.g. insertion of a peripheral venous access for fluid or medication administration or placing urinary tract catheters).

All 25 participating care facilities with up to 100 residents are located in Aachen, Germany, and its surrounding districts (Städteregion Aachen, Düren, Heinsberg). Some of the facilities are specialized to care for residents with advanced dementia while other nursing homes have a mixed geriatric clientele ranging from predominantly self-caring to permanently bedridden and in need of extensive care.

A list of study locations is available at https://www.ukaachen.de/kliniken-institute/optimal-at-nrw/traeger-partner/.

### Eligibility criteria {10}

The inclusion criteria for study participation are:

- Resident of the participating nursing homes
- Age of majority (in Germany: 18 years)
- Valid health insurance status
- Written declaration of consent or written declaration of consent of the legal guardian of residents who are unable to give consent

The exclusion criteria are:

- Persons placed in an institution by order of the authorities or the courts and persons who are in a dependent or employment relationship with the investigator
- No valid health insurance
- Uncertain legal capacity of the person

#### Nursing homes

Eligibility criteria for the participating nursing homes include the usage of the project’s own central electronic health record for documentation purposes and the use of the technical equipment provided. The technical requirements for the use of this equipment must be met (e.g. a wireless network connection). Due to the deployment of non-physician practice assistants, a maximum travel time of approx. 45 min is another criterion for the selection of the participating nursing homes.

#### Non-physician practice assistants

The non-physician practice assistants are qualified with a degree as a medical assistant or nurse. Special activities, such as placing a venous access or the insertion of a urinary catheter, are intensively trained before the start of the intervention phase.

A good command of the German language is a prerequisite, especially when communicating with old people and those who may suffer from dementia.

All non-physician practice assistants are trained in conducting teleconsultations prior to the intervention.

#### Telephysicians

The teledoctors’ main areas of experience are general medicine, geriatrics and emergency medicine. They are intensively trained in the use of telemedical equipment for carrying out teleconsultations.

### Who will take informed consent? {26a}

Informed consent is obtained by investigators trained in Good Clinical Practice (GCP). For this purpose, written patient information (brochures and handouts) and consent forms are distributed, which provide a comprehensive overview of the study contents. Furthermore, information sessions including oral presentations and film sequences illustrating the aim and execution of the trial are held in front of the individual or groups of residents, relatives and legal guardians.

### Additional consent provisions for collection and use of participant data and biological specimens {26b}

With written consent to participate in the study, permission is also granted to access secondary claims data from the statutory health insurance companies in order to increase the data quality of the primary data collected and thus the validity of these data.

## Interventions

### Explanation for the choice of comparators {6b}

Optimal@NRW extends the current standard medical care by including telemedical acute care for geriatric nursing home residents. The intervention is studied under real-world conditions where the intervention is compared to the standard care in nursing homes. The stepped-wedge design ensures that each nursing home is scientifically accompanied and collects data both in the initial regular care operation — the control phase — and later in the intervention phase — i.e. with the use of telemedicine. The control phase serves to record the current state, especially for evaluating the length of stay in the hospital (primary outcome parameter) before the implementation of telemedicine vs. the length of stay after the implementation of teleconsultations and the early warning system (EWS) in the intervention phase.

### Intervention description {11a}

The 25 participating nursing homes are randomized into four clusters by an independent evaluator. A control phase lasting a minimum of 3 months is followed by a 3-month transition phase which in turn is followed by the intervention phase. Depending on the allocated cluster, the various nursing homes start the transition and the following intervention phase at different points in time, whereby the starting points are staggered 3 months apart (e.g. cluster 1: transition phase month 7–9, intervention phase month 10–12 vs. cluster 2: transition phase month 10–12, intervention phase month 13–15).

The German healthcare system consists of outpatient care provided by the Association of Statutory Health Insurance Physicians (ASHIPs), emergency services and inpatient care (hospitals). All three columns of medical care (sectors) are organized and financed differently so that in practice these three sectors work mainly separately. Until today, intersectoral care has not yet been established in Germany. Optimal@NRW strives for an intersectoral form of care by introducing three levels of intervention in parallel within each cluster:

1. Implementation of a new telemedicine approach into the medical supply network by offering a “virtual hub,” which includes:
  Standardized assessment via SmED (“Standardized initial medical assessment for Germany”) by a specialized dispatcher according to this study when a participating nursing home calls the medical emergency service NRW (*Arztrufzentrale NRW 116117*)
  Consecutive determination of urgency and next-level treatment: emergency service (with direct transfer of dataset by prior implementation of new interface), hospital admission, resident health service and telemedicine
  Activation of non-physician practice assistance
2. Opportunity of informed patient-to-physician communication by telemedical consultation:
  Every participating nursing home is equipped with telemedical roller stands: bilateral communication via a high-resolution camera interface, measurement of vital signs (respiratory rate, blood pressure, heart rate, peripheral oxygen saturation), “digital” auscultation and 12-channel ECG
  Telephysician with specialized geriatric expertise available 24/7/365; furthermore, GPs and physicians of the ASHIPs can register for the study and use the telemedical equipment for teleconsultations as well
  Implementation of an electronic health record with collection of patient information (including previous measurements and medical documentation, as well as relevant documents as patient decree/healthcare power of attorney to assure informed decision-making for all people involved) that can be accessed by all study-related telephysicians and registered ASHIP physicians
  Determination of further treatment: acute admission, treatment by GP at nursing home, delegation of treatment steps to either nursing home staff (e.g. application of medication, re-consultation within next hours) or non-medical practice assistance (e.g. administration of IV fluid)
3. Implementation of an early warning system in order to avoid critical health-related situations:
  Regular measurement of vital signs (daily routine) and clinical aspects by nursing home staff
  Transfer of these data into the electronic health record
  Continuous software-based assessment of parameters and transmission to the telemedicine centre
  In cases of potential threatening changes, early alert and activation of newly implemented intersectoral treatment approach coordinated by the telephysician

### Criteria for discontinuing or modifying allocated interventions {11b}

There are no conditions that necessarily lead to discontinuation or modification of the intervention on participant’s level.

At any time, individual withdrawal of informed consent (expressed by the participant or legal guardian) or moving from the participating nursing home leads to termination of study participation. Furthermore, study termination for each participating resident is induced as a care facility discontinues project contribution.

In case of occurring serious adverse events (SAEs), a Safety Board will be activated and finally decides on probable discontinuation and modification of interventions, respectively (see the “Adverse event reporting and harms {22}” section for details).

### Strategies to improve adherence to interventions {11c}

The study will be conducted in accordance with the approved study protocol version, the ICH-GCP (International Conference on Harmonization of Technical Requirements for Registration of Pharmaceuticals for Human Use - Good Clinical Practice) principles, the Declaration of Helsinki, regulatory authority requirements and the standard operating procedures (SOPs) of the project.

Prior to the start of the study, one or more monitoring visit(s) take place to verify and clarify the prerequisites.

Meetings will be held one to two times per week to realign protocol adherence. Regular monitoring visits with spot checks of data entries are intended to uncover problems, reduce discrepancies and verify that data collection and documentation are in accordance with the ICH-GCP principles, the study protocol and the requirements of the regulatory authority.

There is a regular exchange with the independent reviewer (Chair of Health Economics at Bielefeld University) to ensure compliance with the study design.

To maintain proper study-related communication with all partners especially during the Corona pandemic, regular video conferences are held to promote dialogue, sharing of information about ongoing developments within the project and mutual exchange of experiences. Weekly coordination meetings are held with the technology providers and special issues addressed by appropriate working groups.

In order to encourage the nursing home staff to utilize the telemedicine system and proper use of the early warning system, two technology workshops are conducted during the transition phase in each participating nursing home.

In order to improve the cooperation with the participating care facilities, regular on-site visits of the study team and the monitors will take place. Regular standardized trainings of participating partners also take place to ensure proper use of the study’s own technology and database.

The accessibility of the study team for all partners is guaranteed via a uniform telephone hotline and email address.

### Relevant concomitant care permitted or prohibited during the trial {11d}

N/a because intervention means additional aspects to standard care.

### Provisions for post-trial care {30}

After trial completion, the participating nursing home residents will receive the same established standard care as it was provided before the start of the trial. However, if the stakeholders agree and financing can be secured, the Optimal@NRW consortium will try to implement the provision of telemedical care into standard care as the hypotheses of the trial can be confirmed.

### Outcomes {12}

#### Primary outcome

The primary objective of the trial is to evaluate the length of stay in the hospital (including same-day discharge) of inpatient nursing home residents in the intervention versus control cohort. It is hypothesized that the intervention leads to a significant reduction in hospital admissions and thus less days spent in the hospital. This might not only be relevant on the individual patient level (e.g. reduced risk of hospital-acquired infections or other complications such as the development of delirium) but also from the health economic perspective with an assumed improvement of the cost-effectiveness ratio.

#### Secondary outcomes

Two secondary outcomes shall be assessed by the trial to account for the heterogeneity of treatment effects. The primary evaluation of the efficacy as well as the health economic aspects will be realized by the School of Public Health (Bielefeld University). The Human-Computer Interaction Centre (RWTH Aachen University) as well as the Institute for the History, Theory and Ethics of Medicine (University Hospital RWTH Aachen) is responsible for the secondary evaluation strand on the acceptance, ethics and usability of telemedicine in the nursing home setting.

##### Clinical outcome measures

The following parameters related to hospital admissions will be assessed during the study period in the control versus intervention cohort:

- Overall number of hospital admissions
- Hospital admissions grouped by primary diagnosis and leading symptoms, respectively (pneumonia, congestive heart failure, urinary tract infection, delirium, dyspnoea, chest pain, fever, pain, change of consciousness, hypo-/hyperglycaemia, fall, other emergencies)
- Length of ICU stay
- Days spent in the nursing home
- Number of ambulatory care-sensitive conditions
- Number of emergency calls and utilization of ambulance service: paramedic/ambulance, EMS physician (ambulance/helicopter), patient transport ambulance
- Safety of medication (doubling of prescriptions, potentially inappropriate medication according to PRISCUS list 2010)
- Number of medical outpatient contacts (GP, medical specialist)

In relation to the novel telemedicine structure including the implementation of a central electronic health record, the following parameters will be investigated:

- Number and extent of teleconsultation
- Frequency of (suspected) diagnoses
- Time period from emergency call to physician contact
- Influence of frequent teleconsultations on guideline-directed treatment adherence (e.g. hypertonia, hyperglycaemia)

All data will be collected within the study-specific electronic health record. The primary collected data is complemented and validated by claims data of participating statutory health insurers. Both are collected continuously throughout the entire study period.

##### Patient-reported outcome

**Fig. 2:**
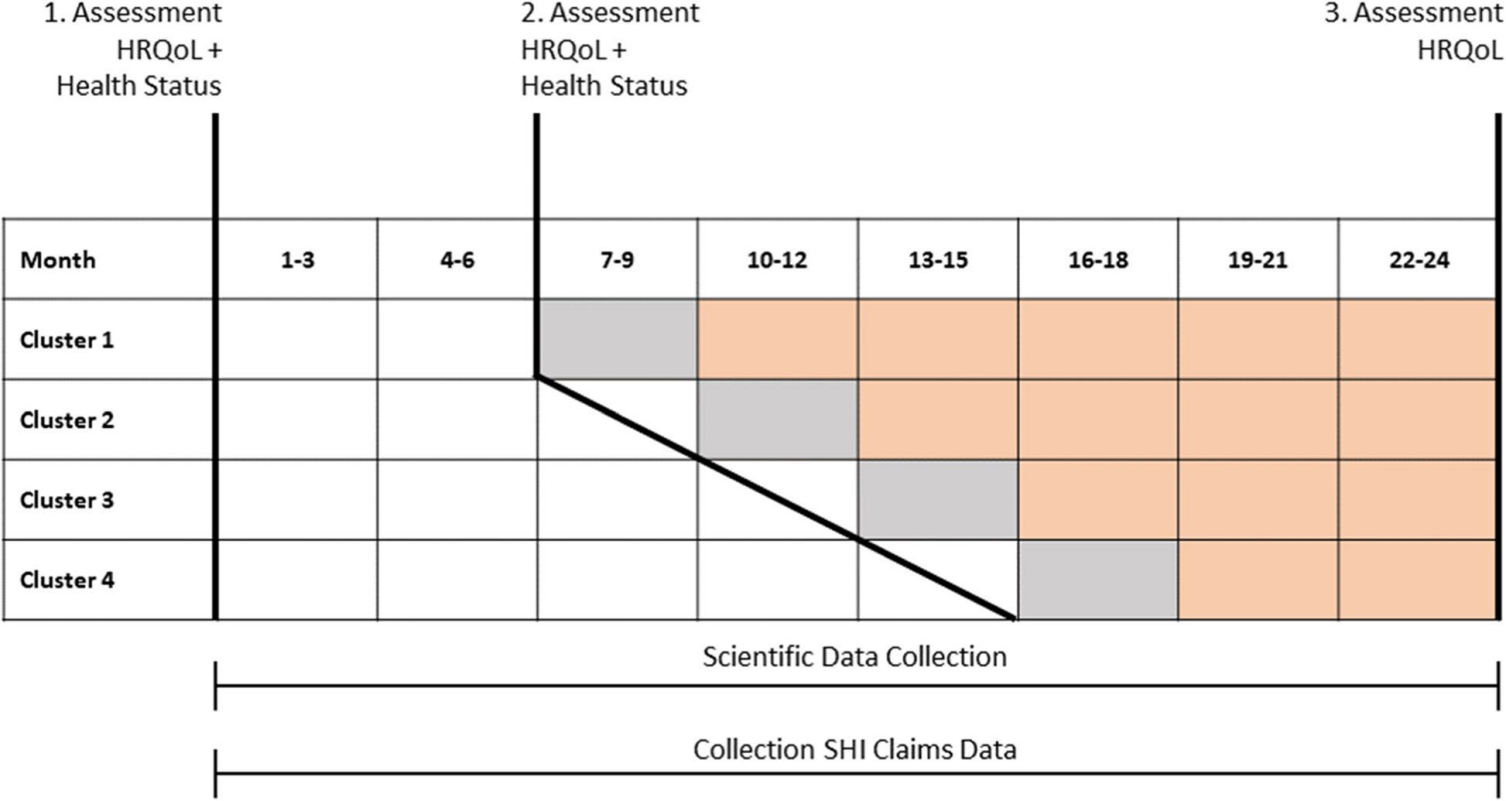
Cluster-based participant timeline

The individual health-related quality of life will be measured at three predefined time points during the entire study period (beginning of the study, beginning of cluster-related transition phase, end of the study; see Fig. 2 in the “Participant timeline {13}” section) by validated questionnaires (VR-12, QoL-AD; detailed information in the “Plans for assessment and collection of outcomes {18a}” section).

##### Health economic outcomes

Additionally, further variables linking economic aspects are assessed based on provided claims data to determine the exhaustive resource utilization of healthcare during the course of the study:

- Healthcare utilization
- Healthcare costs

The effects of the intervention and the costs of healthcare system utilization will be evaluated by a cost-effectiveness analysis to determine the incremental cost-effectiveness ratio (ICER) of an intersectoral telemedicine approach in an acute care setting and to derive conclusions about the cost-effectiveness of the intervention from a financing perspective.

##### Acceptance, ethics and usability of telemedicine approach

Due to the novel approach of telemedicine in a vulnerable group of geriatric patients, the ethical acceptability and the specific demands and needs of this special group will be assessed. The findings shall help to develop a needs-focussed, socially acceptable and sustainable transformation of acute geriatric care at the interface of the nursing home, GP, emergency service and emergency department/hospital. The evaluation is based on questionnaires and structured interviews with nursing home staff and nursing home residents as well as observations by an embedded researcher with focus on the following parameters:

- Current status of healthcare implementation and related problems in daily life
- Communication issues with physicians and care staff
- Intersectoral information transfer including aspects of patient’s autonomy and data privacy
- Individual perception of telemedicine approach and probable related problems
- Evaluation of telemedicine approach based on ethical criteria

### Participant timeline {13}

Due to the stepped-wedge, open-cohort design of the study, participants are continuously enrolled from the beginning of the study until the last cluster. Once enrolled, participants and nursing staff are required to fill in questionnaires regarding the health status (Barthel Index, DSS) of the participants and their health-related quality of life (QoL-AD, VR-12) at baseline.

#### Control phase (white)

All participating nursing homes commenced the control phase on May 1, 2021. Baseline data (health status and HRQoL) of the currently enrolled participants are collected for the control phase and training of the documentation staff is started.

#### Transition phase (grey)

The nursing homes of the first cluster switch to the transition phase on November 1, 2021. The other nursing homes follow according to their cluster after a defined interval of 3 months each. During the transition phase, the technical equipment is delivered and the nursing home staff is trained on the teleconsultation and the EWS. Baseline data of the enrolled patients are re-assessed to determine the health status and HRQoL of the intervention cohort.

#### Intervention phase (orange)

The first cluster transfers to the intervention phase on February 1, 2022. The other clusters follow according to the stepped-wedge design outlined above. During the intervention phase, the teleconsultation system is actively used whenever indicated and participants are then treated by the telephysician. Concurrently, measurement of vital signs via the EWS is performed by staff at regular intervals (independent of teleconsultation utilization). At the end of the study, HRQoL data of all participants is collected for the third time.

### Sample size {14}

In accordance with the primary outcome, sample size calculation is based on the average length of stay in a geriatric cohort in need of care (13.22 days per quarter in residents >70 years) [10] for the control cohort, while a decrease of 30% in days spent in hospital is assumed due to the intervention. For calculation, a two-sided power of treatment effect is considered and a Gaussian distribution of the primary outcome is assumed. Due to the cluster randomization and related organizational considerations, a stepped-wedge design of four clusters with 25 nursing homes was implemented. Applying a linear regression model with an alpha error of 0.05, a power of 1−*β* = 0.90, an assumed standard deviation of 4.5 and an intra-cluster correlation (ICC) of *p*=0.12, 43 participants per nursing home and interval are required to be enrolled. By applying a drop-out rate of 12% per quarter as a consequence of expected high mortality within a sample of nursing home residents [9], the number of participants increases to 49 residents per nursing home and interval. In sum, a sample size of 2.184 participants is estimated. To account for internal validity, the number of participants in each cluster, nursing homes and study phases need to be equally distributed. Sample size calculation is carried out by a health economist at Bielefeld University using R Studio.

### Recruitment {15}

The recruitment of participants takes place on-site at the participating nursing homes. The project was presented to the nursing home managers and their staff as well as the nursing home residents by members of the study team. For the latter group, appropriate language and supporting use of visual media was utilized to achieve a high rate of interest and acceptance. In nursing homes specialized in care of residents with dementia and therefore, a high number of potential participants who are incapable of giving consent, the project was presented to the residents’ legal guardians.

Ongoing recruitment is necessary due to an expected high drop-out rate within a geriatric sample of nursing home residents [9] and in line with the open-cohort sampling design of the study. This will be achieved by continuously informing new nursing home residents via brochures and handouts and regular face-to-face visits of the study team. Furthermore, the leading GPs of each participating nursing home are contacted and informed about the study, so they can give additional advice to interested residents.

## Assignment of interventions: allocation

### Sequence generation {16a}

Due to the study design, the crossover of clusters from control to intervention phase is randomly timed [8] and varies for each unit (i.e. nursing homes) depending on the allocated cluster. Randomization is conducted on the nursing home level before the trial starts by stratification. Nursing homes are randomly allocated to one of four clusters. The following stratification rules are applied:

- Bed capacity
  - ◦ Small entities (< 80 beds per nursing home)
  - ◦ Great entities (> 80 beds per nursing home)
- Presence of specialization in dementia of the nursing home

A random number generator was applied that allows to reproduce the randomization results. Block randomization was done through stratification, where units that are grouped in blocks (stratification arguments) are assigned to the clusters using a complete random assignment within the blocks.

### Concealment mechanism {16b}

To reduce bias of allocation, a concealment mechanism is performed by the independent evaluators of the project that are not involved in recruitment, data collection and intervention. The final list of cluster allocation is provided via mail to the principal investigator in line with the data protection requirements. There is no allocation to interventions, as each resident receives the same interventions and begin of intervention is determined by cluster allocation. An exception is the biosensors of the EWS, which can only be used in some of the nursing homes due to practical considerations.

### Implementation {16c}

Eligible nursing homes are selected by the principal investigators. The health economists from Bielefeld University (not involved in recruitment, data collection and intervention) design the allocation plan. Due to technical and organizational necessities, nursing home managers are informed about the allocated cluster in a specific meeting before the trial starts by the principal investigator. Enrolment of individual participants within nursing homes is managed by the study nurses creating a digital representation of the respective participant in the central electronic patient record, as soon as the signed consent form is available.

## Assignment of interventions: blinding

### Who will be blinded {17a}

Blinding is not applicable as the intervention is implemented in all participating nursing homes with visible differences in healthcare delivery (teleconsultation system).

### Procedure for unblinding if needed {17b}

N/a because of the non-blinded study design

## Data collection and management

### Plans for assessment and collection of outcomes {18a}

After the creation of an electronic health record for each participating resident (whose implementation itself is part of the intervention as there is no unique digital structure in Germany providing comprehensive medical information) by the study team, the medical history data is uploaded there by trained documentation staff or the nursing home staff. Training is provided at the beginning of data collection as well as throughout the course of the study when necessary to ensure maximal data quality. Primary data includes, amongst others, important parts of patient history such as previous diagnoses, allergies and current medication. This is consecutively linked to claims data provided by the participating statutory health insurance companies after study completion. The data collected during a teleconsultation (including date, time, reason for the teleconsultation, vital signs, diagnosis and recommendations) are also fed into the electronic health record via the teleconsultation device. These entries can be made by any of the authorized physicians (study-related telephysicians of the University Hospital RWTH Aachen, registered GPs and ASHIP physicians).

Teleconsultations are intended to be standardized, supported by symptom- or working diagnosis-based standard operating procedures. This ensures high-quality medical care as well as data collection for scientific purposes (evaluation of guideline adherent treatment).

In addition to medical care data, quality of life questionnaires will be collected as patient-reported outcomes (PRO) to allow for comparability and characterization of nursing home residents. Two specific questionnaires are used: VR-12 (Veterans RAND 12-Item Health Survey) for patient-reported health-related quality of life (HRQoL) in the German validated translation [11] and QoL-AD for determining the quality of life of residents suffering from dementia [12]. Both the VR-12 and the QoL-AD are applied to all residents, regardless of the (suspected) presence of dementia.

The instruments are applied at baseline, at the beginning of the control phase or at any time of enrolment, at the beginning of the transition phase and at the end of the intervention phase (please see also Fig. 2 for illustration of time points). This is done in order to have information about the patient cohort available for both the control and intervention phases as well as to evaluate the impact of the intervention on the HRQoL as another secondary endpoint.

In order to adequately account for manifold patient characteristics in the statistical models, additional instruments are used to measure health status.

The Barthel Index in the modified version according to the Hamburg Classification Manual surveys the activities of daily living (ADLs) in a standardized and validated manner [13]. The questionnaire is filled out by the patients themselves as well as by nursing staff.

The Dementia Screening Score (DSS) is used to identify participants with dementia in inpatient geriatric care by means of third-party assessment (i.e. nursing staff) [14].

Retrospectively, routine health insurance data are used to determine the weighted Charlson comorbidity index [15]. By using the weighted approach introduced by Quan et al. [16], it is possible to use the billing data of the statutory health insurances (SHI).

Data quality is ensured through proper training and close external monitoring. In addition, GCP-compliant documentation that is meaningful in terms of content is monitored. If necessary, training sessions are repeated.

After the completion of the intervention phase, the collected medical data are transferred to a study database (electronic case report form (eCRF)) for scientific analyses (further details are mentioned in the “Data management {19}” section).

### Plans to promote participant retention and complete follow-up {18b}

No specific activities related to participant retention are planned because the intervention represents an additional opportunity to standard treatment of outpatient medical care. The decision to initiate medical consultation is primarily taken by nursing home staff and the dispatcher at the medical emergency service NRW. Thereby increasing appreciation amongst nursing home staff is expected to lead to higher acceptance and thus to high utilization of the new telemedicine approach.

Participants may withdraw from the study for any reason at any time. This being said, we expect the most drop-outs will occur due to high mortality within this special group of geriatric patients. In case an individual discontinues participation, the underlying reason (death, withdrawal of informed consent, move to non-participating nursing home, appearance of SAE) will be recorded.

### Data management {19}

All relevant medical and personal data are collected within a central electronic patient record (*zEPA*) during the complete course of the study. Additionally, the paper-based questionnaires measuring HRQoL will be entered into the zEPA as well. This will be performed by specially trained documentation assistants of the participating nursing homes. For study purposes, the data sets are further transferred into an eCRF.

German SHIs are legally obliged to collect and store claims data. Participating SHIs (*Techniker Krankenkasse*, *Barmer GEK*, *DAK*, *IKK Classic*, *AOK*) provide a predefined data set (outpatient and inpatient treatments, prescriptions, therapeutic aids, care level) for each enrolled participant within a predefined time frame (four quarters after study intervention and four quarters prior to study for risk adjustment).

#### Data quality

The data collection is planned and conducted according to the standard operating procedures (SOPs) of the *Centre for Translational & Clinical Research of the RWTH Aachen University Faculty of Medicine* (*CTC-A*). The documentation assistants of the nursing homes, who collect and enter the study-related medical and personal data, are specifically trained by independent monitors of the CTC-A to acquire valid, objective and reliable data sets. The training is conducted prior to the beginning of the trial and additional trainings will take place as needed. Furthermore, data entries will be completed and checked by two qualified study nurses of the *CTC-A*.

The primary data as well as the claims data will undergo a plausibility assessment by the independent evaluation institution (*University of Bielefeld*) before a detailed analysis is conducted. In case of major implausibility within the data, feedback to review data quality will be given via the trusted third party (TTP).

#### Data flow and security

To investigate the primary and secondary objectives of the trial, separate data sets are needed: firstly, study-related personal and medical data and secondly claims data from SHIs. Proper data management and transfer according to current regulations must be ensured between the following relevant institutions: study sites (participating nursing homes), trial centre (*University Hospital RWTH Aachen*), independent evaluation institution (*University of Bielefeld*) and participating SHIs.

A data protection policy in agreement with the general data protection regulation is implemented by involving an independent TTP that supervises the identity management and provides the infrastructure for a two-stepped pseudonymization, linkage and transfer of data sets. The TTP has no access to medical data. Data collection and consent management are located within the participating study sites (detailed description in the “Confidentiality {27}” section).

Access to unencrypted data is restricted to authorized personnel only in order to verify the proper conduct of the trial.

The participants are informed about the data processing and protection management in the trial information material.

#### Data storage

The investigator will retain all trial-specific data (electronic and paper-based source documents, e.g. questionnaires, written consent) in accordance with local requirements and premises. After completion of the trial, proper storage and administration of all trial documents will be ensured for at least 10 years in accordance with the statutory provisions. Deletion will take place no later than 31.12.2034. After this date, personal data will be deleted, unless legal, statutory or contractual retention periods conflict with this.

The patient list maintained by the TTP is available only until the end of the trial (30.04.2024). From this date onward, no re-identification based on personal data will be possible anymore.

### Confidentiality {27}

The individual data, which are collected within the *zEPA* under a patient ID (*IDAT with PID*), are split into medical and identifying data. Identifying data are transferred in batches to the TTP, implementing the first step of pseudonymization (*PSN1*; in accordance with Art. 4 Nr. 5 GDPR) and maintaining a patient list for probable re-identification. The necessary pseudonyms are routed via the TTP, while study-related medical data (*MDAT*) are sent directly to a trial database for research-specific storage. Every batch of data can be linked via a batch ID and the respective index. At the end of the trial, all data are transferred in the same way to the independent evaluation institution, which, thus, has access to data under a second pseudonym (PSN2). Any other extraction of data from the trial database for research purposes has to be anonymized and approved by a Data Use and Access Committee (DUAC).

Claims data are collected and stored according to the regulations of the participating SHIs that will be queried for the enrolled participants by the TTP (identified by the health insurance number). Utilization of those claims data while guaranteeing data protection according to §75 SGB X is verified by an application submitted to the Federal Office for Social Security (*Bundesamt für Soziale Sicherung*).

**Fig. 3:**
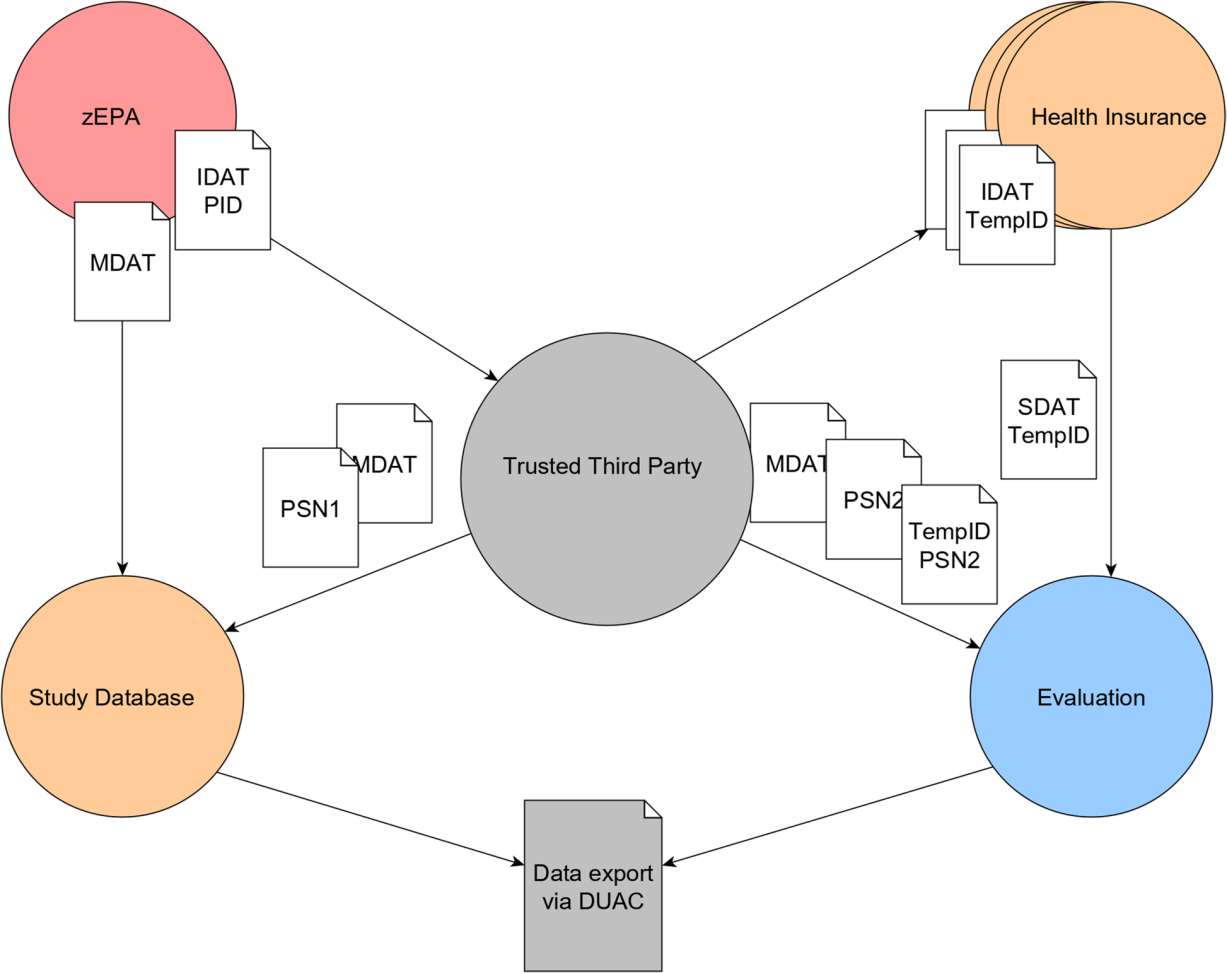
Data flow concept

The SHIs provide the data set (*SDAT*) requested by the TTP with a temporary ID (TempID) and the respective health insurance number. Data are provided in a pseudonymized manner (*TempID*) to the independent evaluation institution. Furthermore, the evaluation institution receives the two-staged pseudonymized data set *(lookup table TempID to PSN2*) from the TTP and the study-related medical data (*MDAT labelled with PSN2*) from the trial database. Now, a linkage between claims data and study-related data can be realized and further analyses for investigating primary and secondary objectives will be conducted (please see also Fig. 3 for visualization).

The concept of scientific data management of the project was developed by the *Institute of Medical Informatics of the University Hospital RWTH Aachen* and is available on request (German language only). This approach implies that no participating institution has an insight into the personal and medical data (study-related and claims data) at the same time. Thus, a high standard of data protection related to personal data as stated in the GDPR will be achieved.

### Plans for collection, laboratory evaluation and storage of biological specimens for genetic or molecular analysis in this trial/future use {33}

N/a because no biological specimens are collected.

## Statistical methods

### Statistical methods for primary and secondary outcomes {20a}

First, sample characteristics are summarized using descriptive statistics like absolute and relative frequency distributions as well as dispersion (e.g. median, mean, quantile, variance or standard derivation). To identify group differences between study phases, control and intervention groups are compared regarding their primary and secondary outcomes by suitable statistical tests, e.g. for normally distributed continuous variables *t*-tests (otherwise Wilcoxon rank-sum tests) or chi-square tests for categorical variables. For the latter, if there are less than five observations in minimum of one cell of contingency tables, Fisher exact tests will be applied.

Primary and secondary outcomes are further evaluated by inferential statistical analysis using appropriate statistical methods (e.g. Mann-Whitney *U*-test for independent samples without normal distribution or Fisher exact test for binary outcomes). Data of the integrated transition phase is not considered in the evaluation. To assess the influence of regressors on the primary endpoint, a suitable regression model is applied. Hereby, a generalized linear regression model is anticipated to be estimated, where the length of stay (dependent variable) is considered as (zero inflated) Poisson- or negative-binomial distributed variable in the model. Due to time as a possible confounder in stepped-wedge studies, secular trends will be addressed in the applied regression model as an independent variable [17]. Furthermore, the respective model will control for possible confounders like age, sex, grade of multimorbidity or non-observed characteristics of the patients, the nursing homes or hospitals. If applicable, suitable regression models will be further carried out for relevant secondary outcomes similar to the analysis strategy of the primary endpoint. The results of all models are tested regarding their validity by using a residuals analysis. All statistical analyses are based on a significance level of 5%. Data preparation and evaluation are performed by health economists at Bielefeld University using R and suitable packages.

### Methods for additional analyses (e.g. subgroup analyses) {20b}

Subgroup analyses for the primary endpoint are applied to analyse potential influences on the effect and to test the robustness of study results, in terms of participant characteristics (e.g. age, sex), intervention aspects (early warning score, teleconsultation) and data bases (e.g. longitudinal vs. cross-sectional data, electronic health record data vs. claims data). These additional analyses will be evaluated using the same statistical methods as described above. Furthermore, besides evaluation over the entire cohort, analyses between clusters are carried out to evaluate the structure of data and its effects on the primary outcome.

Additionally, an economic evaluation of the intervention will be conducted to determine the incremental cost-effectiveness ratio (ICER). It is defined by dividing the difference of costs (nominator) and intervention effect (denominator) between the control and intervention phases. If at least one effect (cost, outcome) is determined as significant or a trend of cost-effectiveness (less costs or more effectiveness) can be recognized, the cost-effectiveness model will be tested regarding its robustness. A probabilistic sensitivity analysis will be carried out using a bootstrapping technique with *n* = 500 random samples, and the parameters of the cost-effectiveness model will be calculated again for each sample. Results of it will be presented in a cost-effectiveness plane. The cost-effectiveness model is performed from a third-party payer perspective following the recommendations of the German Institute for Quality and Efficiency in Health Care (IQWiG) [18]. The economic evaluation is in line with the principles of good secondary data analysis [19], the recommendations of the memorandum “Methods for Health Services Research” [20] and the standards of the German Evaluation Society [21].

### Methods in analysis to handle protocol non-adherence and any statistical methods to handle missing data {20c}

Adherence of the nursing homes to apply the intervention is checked through descriptive statistics based on the electronic health records data, where the number of teleconsultations is operationalized as a surrogate for adherence. The applied regression models will control for specific nursing home characteristics as interdependent variables of the equation including adherence to the intervention,

As an intention-to-treat analysis of data is anticipated for evaluation, all included participants are considered in the analyses. The plausibility of data will be checked in advance. Missing and censored data are tested regarding their structure, e.g. their occurrence (random or non-random) and their absolute/relative frequencies. If these data occur randomly and the number of missing/censored data is relatively large limiting the study results significantly, imputation mechanisms are considered to apply. Possible methods encompass regression imputation or mean imputation. Results of models using imputed data are presented in comparison with the same models without imputed data to increase the validity of the method chosen and the transparency of the results. A sensitivity analysis following a complete case principle will also be performed.

### Interim analyses {21b}

N/a because no interim analyses are intended.

### Plans to give access to the full protocol, participant-level data and statistical code {31c}

The full protocol is available as a Supplementary 1. Non-identifiable participant-level datasets might be available on request to the principal investigator.

## Oversight and monitoring

### Composition of the coordinating centre and trial steering committee {5d}

The study team of the coordinating centre (PI, medical trial managers, study physicians, data manager, administrative team) meets four times a week to coordinate on current and forthcoming tasks and issues concerning trial execution. Once a week, the study team meets with representatives of the technical provider to consider ongoing developments and potential changes of the technical equipment. Additionally, the whole study team meets once per month to review the current trial status according to a preliminary consented Gantt chart.

The administrative project team coordinates the interaction of all stakeholders by scheduling monthly jours fixes and manages all regulatory issues.

The medical members of the project team take care of ongoing recruitment, teleconsultations, interaction with the nursing homes, interaction with the monitoring team and training of the non-physician practice assistants.

Two external monitors of the CTC-A are responsible for overseeing appropriate data collection in the participating nursing homes as well as GCP conform data management within the study. For this purpose, bi-weekly meetings are scheduled with the project team.

The overall project is evaluated by an independent evaluator (Faculty of Health Sciences at Bielefeld University) and consulted at least once a month on the current status of the trial.

### Composition of the data monitoring committee, its role and reporting structure {21a}

A Data Monitoring Committee (DMC) is not necessary according to current recommendations (including FDA/CBER, 2006: DMC needed in case of blinding, established study discontinuation criteria, existing safety concerns, etc.) [22].

A Data Use and Access Committee (DUAC, see the “Confidentiality {27}” section) has been created to govern decisions about approval of data extraction from the research database for scientific purposes other than the main evaluation.

### Adverse event reporting and harms {22}

Patient safety is supervised and assessed by a Safety Board.

It consists of three independent members who are not involved in the implementation of the project. The Safety Board is alerted in case of (serious) adverse events (SAEs) and decides on trial discontinuation for individual participants if patient safety is compromised. The Safety Board can also encourage a change in interventions. The following points count as SAEs:

- Resuscitation during teleconsultation
- Unexpected death during teleconsultation
- Unexpected death within 24 h of a teleconsultation
- Unexpected hospitalization within 24 h of a teleconsultation
- Unexpected death while wearing a biosensor (if available)

Adverse events or serious adverse events are documented, evaluated and reported in accordance with GCP and the Professional Code of Conduct for Physicians (BOÄ, *Berufsordnung für Ärzte*).

### Frequency and plans for auditing trial conduct {23}

This trial will be reviewed by qualified monitors of the *CTC-A* in accordance with the current ICH-GCP guidelines. All related activities are predefined within a *Monitoring Manual* (available on request).

The monitors perform regular visits to the nursing homes prior to and during the trial period in order to detect and eliminate shortcomings. Furthermore, the main site is systematically checked focussing on verification of informed consent forms (ICFs) and essential documents. All staff members support the monitors and provide all necessary information including access to the original data.

The processes listed below will be reviewed during those monitoring visits:

- Adherence to the study protocol, the ICH-GCP principles, the Declaration of Helsinki and any according regulatory authority requirements
- Integrity of source data and eCRF entries
- Accuracy of ICFs
- Correct documentation and reporting of SAEs
- Review of relevant trial-related logs (e.g. screening and enrolment log, study staff log, monitoring log)
- Accuracy and completeness of the Trial Master File (TMF)

### Plans for communicating important protocol amendments to relevant parties (e.g. trial participants, ethical committees) {25}

Any changes to the protocol, the informed consent form and/or information provided to study participants will be coordinated with the independent evaluator. Subsequently, the changes are submitted to the responsible ethics committee for review and approval prior to implementation (exception: logistical or administrative changes or to avoid direct hazards).

Any amendment to the protocol will be signed by the principal investigator (PI).

In addition to the approval of the responsible ethics committee, amendments also require the approval of the sponsor (German Aerospace Centre, *Deutsches Zentrum für Luft- und Raumfahrt (DLR)*) prior to implementation.

According to the regulations of the consortium, the partners of Optimal@NRW will be informed in writing afterwards. Should a relevant change affect the consent form, the study participants will be informed in writing. Where necessary, the written consent of the participants will be renewed. This is done in close coordination with the responsible ethics committee.

Additionally, the Optimal@NRW Steering Committee can be called in to coordinate and agree on the required steps with the partners involved.

## Dissemination plans {31a}

The findings of the trial will be disseminated by publication of manuscripts in peer-reviewed scientific journals and by conference contributions (abstracts, posters or talks).

The responsible ethics committee will also be informed of the trial results.

Furthermore, the results will be discussed with clinical experts and leading authorities of the SHIs and the ASHIPs in order to implement the telemedicine approach into standard care.

## Discussion

Optimal@NRW creates a new intersectoral approach of telemedical care. Cooperation between general practitioners, out-of-hour healthcare services, hospitals and emergency services has not been implemented that way in Germany yet.

During the planning phase, many interesting aspects have already become apparent, some of which are highly relevant for a later incorporation into standard medical care. Currently, there are some limitations concerning effective communication with the rescue coordination centres. For example, the digital interface between the doctors’ call centre and the emergency service coordination centres established in the project is still in many ways restricted (due to e.g. data protection requirements) and should be significantly expanded in the future. Additionally, there is still no interface between the telemedicine centre at RWTH Aachen University Hospital and the emergency service coordination centres. This will be of significant relevance for consultations during which the telephysician identifies the need to alert the emergency services and responsible emergency service coordination centre.

Timely access to medication for the treatment of an acute medical condition represents a grave care gap for which the current healthcare system has no applicable solution. At present, the German Pharmaceuticals Act prohibits nursing homes to stockpile medications which have not been previously prescribed for a patient. If a nursing home resident requires medication outside regular practice and pharmacy opening hours, there are currently no regulations governing either the prescription of the medication by a telephysician or the delivery of the medication to the nursing home. In the future, a telemedical infrastructure will simplify the process of prescribing medication for nursing home residents. However, the delivery of the medication must also be ensured outside regular pharmacy opening hours. Optimal@NRW will initially achieve this by dispatching the non-physician practice assistance to nursing homes in need of assistance. During the course of the trial, the frequency, type and impact of telemedically prescribed medication will become apparent, thereby documenting the potential for this new kind of medical care.

The novel kind of intersectoral care being provided by this project establishes innovative forms of doctor-patient communication and ways to prescribe and deliver medication in an acute medical setting. It should also help to provide a starting point from which to clarify and streamline the regulations governing these processes. Above all else, it establishes an intersectoral care authority which can provide qualified help and personnel on-site in a timely manner. This can at least partially compensate for the current deficit in remote care.

A training curriculum for so-called non-physician practice assistants already exists at the German Medical Association. Modification of the curriculum to include skills specific to the delivery of telemedical care, as they are required for Optimal@NRW, has not yet been considered. Optimal@NRW will investigate and define the skills required by non-physician practice assistants in order to develop a proposal for modifying the training curriculum.

When contemplating the considerable potential benefits telemedical care can bring, one must also consider the large challenges still ahead: The capability and extent of implementation of digital care software used for, amongst other things, documentation in nursing homes is heterogenous. As of now, there is still no uniform interface which could facilitate seamless communication between telemedical and nursing home software systems. On top of this, deficient network quality within nursing homes but also around them in rural areas will make the provision of telemedical care, which requires broadband speeds, a challenge. But if these obstacles can be removed, the intersectoral and telemedical approach to health care holds great promise for patients and society alike.

## Trial status

- Start of recruitment: 05/03/2021
- Nursing homes of the first cluster in the intervention phase, the second cluster in the transition phase, third and fourth clusters in the control phase
- Recruitment is ongoing up until 04/2023 due to the open-cohort stepped-wedge cluster design
- Protocol version 02, 09/03/2021

## Supplementary Information

**Additional file 1.**

## Data Availability

The final dataset for evaluating primary and secondary outcomes will be accessible by the responsible employees of the University of Bielefeld after a two-step pseudonymization via the TTP. The study database including medical data derived from the trial can be accessed by the Optimal@NRW Research Group for specific research purposes after receiving approval by the Data Use and Access Committee of the trial.

## Abbreviations

ACSH: Ambulatory care-sensitive hospitalization
ADLs: Activities of daily living
AE: Adverse event
ASHIP: Association of Statutory Health Insurance Physicians
BOÄ: Code for professional conduct for physicians *(Berufsordnung für Ärzte)*
CTC-A: Centre for Translational and Clinical Research Aachen
DMC: Data Monitoring Committee
DSS: Dementia Screening Score
DUAC: Data Use and Access Committee
eCRF: Electronic case report form
FDA: Food and Drug Administration
GDPR: General Data Protection Regulation
GP: General practitioner
ID: Identification
IDAT: Identifying data
ICF: Informed consent form
ICH-GCP: International Conference on Harmonization of Technical Requirements for Registration of Pharmaceuticals for Human Use - Good Clinical Practice
HRQoL: Health-related quality of life
MDAT: Medical data
PI: Principal investigator
PID: Patient ID
PRO: Patient-related outcome
PSN1, PSN2: First/second pseudonym
QoL-AD: Quality of Life in Alzheimer Dementia
SAE: Serious adverse event
SDAT: Statutory Health Insurance Data
SGB X: Code of Social Law (*Sozialgesetzbuch X*)
SHI: Statutory Health Insurance
SOP: Standard operating procedure
TMF: Trial Master File
TTP: Trusted third party
VR-12: Veterans RAND 12-Item Health Survey
zEPA: Central Electronic Patient Record (German: zentrale Elektronische Patienten Akte)
