# Supplementary material for: Optimal@NRW: optimized acute care of nursing home residents using an intersectoral telemedical cooperation network — study protocol for a stepped-wedge trial
Source: Trials. 2022 Sep 27;23:814. doi: 10.1186/s13063-022-06613-1 (PMC9513974; doi:10.1186/s13063-022-06613-1)
Supplement: Supplementary file 1 — Additional file 1. [file 13063_2022_6613_MOESM1_ESM.pdf]

Study Protocol

Optimised acute care of geriatric patients using an intersectoral telemedical cooperation network - around the clock*

Optimal@NRW

***) Original title: Optimised acute care of geriatric patients using an intersectoral telemedical cooperation network - around the clock**

**Trial number**	*19-019*
**Project Manager/ Principal investigator**	**Priv.-Doz. Dr. med. Jörg Christian Brokmann**<br>Head of the Central Emergency Department<br>RWTH Aachen University Hospital
**Co-investigator**	**David Brücken, MD**<br>**Christian Hübel, MD**<br>Senior Physicians<br>Central Emergency Department<br>RWTH Aachen University Hospital
**Status, version, date**	Final, V03, 15.02.2022

Auditors and Administrative Structures

**Head of clinical trial/Investigator**	Priv.-Doz. Dr. med. Jörg Christian Brokmann<br>Head of the Central Emergency Department<br>Central Emergency Department<br>RWTH Aachen University Hospital<br>Pauwelsstr. 30<br>52074 Aachen, Germany<br>T+49 241 80 80804<br>F+49 241 80 33 80804<br>E-Mail: jbrokmann@ukaachen.de
**Co-investigator**	**Dr. med. David Brücken**<br>**Dr. med. Christian Hübel**<br>Senior Physicians<br>Central Emergency Department<br>RWTH Aachen University Hospital<br>Pauwelsstr. 30<br>52074 Aachen, Germany<br>T+49 241 80 80804<br>F+49 241 80 33 80804<br>E-Mail: dbruecken@ukaachen.de & chuebel@ukaachen.de
**Statistics of the medical questions**	Prepared by investigators and co-investigators
**Evaluation of the overall trial and statistics of the health economic questions**	Prof. Dr. Wolfgang Greiner<br>Faculty of Health Sciences, WG 5 - Health Ecology and Health Management<br>Bielefeld University<br>Universitätsstr. 25<br>33615 Bielefeld, Germany<br>Tel: 0521 106 6989<br>Fax: 0521 106 156989<br>E-mail: wolfgang.greiner@uni-bielefeld.de

Investigators and Administrative Structures

**Project management**	Innovation Centre Digital Medicine (IZDM)<br>RWTH Aachen University Hospital<br>Susanne Rademacher, Project Manager<br>Cynthia Wendt, Project Manager<br>Kerstin Voncken, Project Manager<br>Pauwelsstr. 30<br>52074 Aachen, Germany<br>Tel.: 0241 80 88726<br>Fax: 0241 80 33 80804<br>E-mail: optimal-nrw@ukaachen.de
**Monitoring**	Centre for Translational & Clinical Research Aachen (CTC-A)<br>Pauwelsstr. 30<br>52074 Aachen, Germany<br>Tel.: 0241 80 80092<br>Fax: 0241 80 33 80092<br>E-Mail: ctc-a@ukaachen.de
**Ethics Committee**	Ethics Committee of the Medical Faculty of the University Hospital of RWTH Aachen University<br>Chairman: Prof. Dr. Günther Schmalzing<br>Pauwelsstr. 30<br>52074 Aachen, Germany<br>Tel.: 0241 80 89963<br>Fax: 0241 80 82012<br>E-Mail: ekaachen@ukaachen.de

Content

Synopsis 6
Abbreviations 11
1 Rationale and clinical relevance 11
2 Aim of the trial 12
2.1 Primary target parameters 14
2.2 Secondary target parameters 14
2.3 Further target parameters 14
2.4 Hypothesis 15
2.5 Parallel evaluation strands 15
3 Trial design and duration 16
3.1 Trial design 16
3.2 Duration of trial 18
4 Trial population 18
4.1 Number of patients 18
4.2 Inclusion criteria 18
4.3 Exclusion criteria 18
5 Trial treatments and procedure 18
5.1 Project procedure 18
6 Data collection 21
7 Safety data, records and notifications 23
7.1 Safety Board 23
7.2 Documentation of events that could potentially jeopardise patient safety 23
8 Termination of trial 23
9 Further treatment plan 24
10 Statistics 24
10.1 Case number calculation and sample size 24
11 Ethical and legal aspects 25
11.1 Independent Ethics Committee 25
11.2 Information and declaration of consent 25

11.2.1 Patient ID and care record labelling ... 26
11.3 Delegation of investigator duties ... 26
11.4 Trial plan changes ... 26
11.5 Data protection ... 27
11.5.1 Collection, access and use of personal data ... 28
12 Quality assurance and control ... 29
12.1 Documentation of original data ... 29
12.2 Data management ... 29
12.3 Direct access to the original data ... 29
12.4 Monitoring ... 29
13 Handling, documentation and archiving of original data ... 30
13.1 Documentation completion ... 30
13.2 Data correction ... 30
13.3 Archiving of trial documents ... 30
13.4 Destruction of trial documents ... 31
14 Publication ... 31
15 Financing and insurance ... 31
15.1 Funding ... 31
15.2 Insurance ... 31
16 Declaration of conformity ... 31
17 Signatures ... 33
18 Literature ... 34

Synopsis

Item	Description
**Title of trial**	Optimised acute care of geriatric patients using an intersectoral telemedical cooperation network - around the clock
**Short title**	**Optimal@NRW**
**Trial number**	19-019
**Protocol version**	Version 03, 15.02.2022
**Registration**	The present trial was registered at ClinTrials.gov with the identification number NCT04879537 after approval by the responsible ethics committee and before inclusion of the first patient.
**Legislation**	The implementation of the project is the responsibility of the Berufsordnung für Ärztinnen und Ärzte (BOÄ) (professional code of conduct for physicians).<br>The project will be implemented according to the ethical principles originating in the Declaration of Helsinki.
**Trial centre/ consortium leadership**	Trial centre :<br>Priv.-Doz. Dr. med. Jörg Christian Brokmann<br>Central Emergency Department<br>RWTH Aachen University Hospital<br>Pauwelsstr. 30<br>52074 Aachen, Germany<br>Tel.: 0241 80 80804<br>Fax: 0241 80 33 80804<br>Mail: jbrokmann@ukaachen.de

**Consortium partner**	• Kassenärztliche Vereinigung Nordrhein (National Association of Statutory Health Insurance Physicians North Rhine)<br>• Techniker Krankenkasse<br>• Barmer<br>• DAK<br>• IKK classic<br>• Bielefeld University, Faculty of Health Sciences, WG 5 - Health Economics and Health Care Management<br>• Participating nursing homes: see nursing home list

Item	Description
**Funding**	The project is funded by the Innovation Fund of the Federal Joint Committee for the Promotion of New Forms of Care (§92a para. 1 SGB V). The funding period of the full funding starts on 01 April 2020 and ends on 31 March 2024.<br>Funding code: 01NVF19015<br>The details can be found in the funding decision.
**Insurance**	No additional patient insurance has been taken out. In the event of fault-based incidents, patients are covered by the hospital's usual liability insurance.<br>The University Hospital of RWTH Aachen has a public liability insurance policy with Allianz Versicherungs-AG (insurance number: AS-9104141415).<br>A road accident insurance policy has not been taken out.
**Risk-benefit assessment**	**Possible risks**<br>• There are no possible risks for the participants.<br>**Possible benefit**<br>• Participants in the Optimal@NRW project may potentially benefit from faster and more needs-based treatment by using telemedicine.<br>• Hospitalizations for ambulatory care-sensitive conditions (ACSH) can possibly be avoided through the project. Participants in the Optimal@NRW project may benefit from a reduction in dispensable ambulance transports and hospital stays.<br>**Potential benefits for the general public**<br>A possible benefit for the general public is that overuse of emergency departments due to avoidable admissions is avoided and emergency departments are enabled to fulfil their core activities. In addition, it is expected that non-emergency trips by emergency services and the avoidable occupancy of hospital beds will be reduced, thus reducing costs and resources in the health system for the general public.
**Trial rationale**	Optimised health care for geriatric patients by introducing an intersectoral telemedical cooperation network and an early warning system.
**Primary target parameter**	Reduction of hospital days, increase of days spent at home
**Secondary target parameter**	• ICU length of stay<br>• Organ failure

Item	Description
	• Mortality<br>• Hospital length of stay with regard to main diagnosis through telemedical visit after discharge<br>• Number of hospitalizations for ambulatory care-sensitive conditions (ACSH)<br>• 30-day survival/hospital survival<br>• nosocomial infections<br><br>other
**Further target parameters**	• Duration of technology development until first system tests<br>• Scope of consultation via teleconsultation<br>• Reduction in the use of emergency services and emergency admissions through the use of the virtual counter and consequent increase in the use of outpatient care structures (GPs / medical on-call service).<br>• Reduction of incorrect medication<br>• Increasing the general quality of care by reducing the time to contact a doctor and establishing guideline-based treatment.<br>• Concordance rate of prehospital suspected/present diagnoses with the discharge diagnosis after hospitalisation<br>• General improvement of the patient outcome<br>• Effect of regular teleconsultations on guideline adherence for hypertension, blood glucose imbalance, outpatient infections, frailty<br>• Shortening reaction interval until doctor contact<br>• Cost-effectiveness analysis for later expansion of EVA sites<br><br>other
**Trial design**	Multicentre, prospective, cluster-randomised controlled intervention trial in stepped-wedge design
**Duration of trial**	Planned trial start: 15.03.2021<br>Start of data collection: 01.05.2021<br>Active trial duration: 24 months<br>Data analysis: 3 months<br>Total duration of trial: 28 months<br>For the individual patient: depending on the cluster 6-15 months
**Number of patients**	A total of N=2,184 participants will be included in the trial.

Item	Description
**Inclusion criteria**	1. Residents of one of the participating nursing homes.<br>2. Age of majority<br>3. Written declaration of consent<br>4. Consent of the guardian for residents who are unable to consent
**Exclusion criteria**	1. Persons placed in an institution by order of the authorities or the courts<br>2. Persons who are in a dependent or employment relationship with the auditor<br>3. Unclear ability of the resident to give consent
**Methods**	By equipping nursing homes with teleconsultation trolleys and early warning systems and using this innovative teleconsultation infrastructure, unnecessary hospital admissions are to be avoided. The intersectoral approach of the project, which forms a synopsis between the outpatient sector, the inpatient sector and the ambulance service, serves this purpose. This is supplemented by the so-called "virtual digital counter".
**Number of cases and statistical considerations**	The average number of hospital days of people in need of care is 13.16 days per quarter (Jacobs, Kuhlmey & Greß, 2020). Considering the age structure >70 years (Federal Statistical Office, 2019), this results in an underlying average number of hospital days of 13.22 for the defined trial population. For the case number calculation, it is assumed that this can be reduced by 30% to 9.25 days. The assumption of the effect size was quantified by specialists of the University Hospital Aachen. The internal dependence of the individual clusters is estimated with an intra-class correlation coefficient (ICC) of $\rho=0.12$, which is based on the ICC calculation of a preliminary trial by Hoffmann & Schmiemann (2017) for cluster-based studies in nursing home settings as well as the consideration of the trial design with several nursing homes in one cluster. With an α-error of 0.05, a power of $1-\beta = 0.90$ and an assumed standard deviation of 4.5, an underlying linear regression model results in a case number of 43 nursing home residents per nursing home and time interval. Due to the high mortality rate of the trial population (Vossius et al., 2018) as well as other factors of a possible dropout, a quarterly dropout of 12% is assumed, so that the number of cases increases to 49 nursing home residents per nursing home and time interval. This corresponds to an additional quarterly recruiting of approx. 6 nursing home residents per nursing home. This results in a total case number of **N=2,184 persons**. In other words, this means a case number of n=1,173 per interval, which means that N=9,383 observations are available as cross-sectional date for all persons across all clusters, resulting both from longitudinal data of n=1,032 persons and the n=1,152 persons additionally recruited.

Item	Description
	Distributed over the individual phases of the trial design, this results in a number of cases at observation level of n=4,105 in the control group, n=1,173 in the transition phase and n=4,105 in the intervention phase, so that at person level the above-mentioned number of cases of 49 nursing home residents per nursing home and time interval results. To ensure the internal validity of a cluster, it must be ensured that the number of nursing homes and their residents are almost homogeneously distributed.<br><br>The patient data of the control phase will be compared with the patient data of the intervention phase with regard to the primary and secondary endpoints. The collected data are analysed descriptively. Continuous variables are summarised by calculating mean, median, standard deviation and interquartile range. The distributions of the continuous variables are evaluated by means of histograms and boxplots. Frequencies and percentages are calculated for categorical variables. The descriptive analysis is performed both comprehensively for all trial participants and stratified by treatment group.

Abbreviations

AE	Adverse Event
ASK	Ambulatory Care Sensitive Hospitalizations
BDSG	Federal Date Protection Act
BOÄ	Code of Conduct for Physicians
CRF/eCRF	(Electronic) Case Report Form
CTC-A	Centre for Translational & Clinical Research Aachen
EDV	Electronic Data Processing
ECG	Electrocardiogram
EWS	Early Warning System
GCP/GCP-V	Good Clinical Practice/Good Clinical Practice Act
HA	General Practitioner
ICH	International Declaration of Helsinki
IEC	Independent Ethics Committee
IRB	Independent Review Board
ICU	Intensive Care Unit
ISF	Investigator Site File
IZDM	Innovation Centre Digital Medicine at RWTH Aachen University
KTW	Hospital Ambulance Service
KV	Association of Statutory Health Insurance Physicians
LKP	Head of Clinical Trial
LAN/WLAN	(Wireless) Local Area Network
NEF	Emergency Vehicle
RD	Emergency Medical Service
RTW	Ambulance
RTH	Ambulance Rescue Helicopter
RWTH Aachen	Rheinisch-Westfälisch Technische Hochschule Aachen
SAE	Serious Adverse Event
SD	Standard Deviation
SmED	Structured Initial Medical Assessment in Germany
SOP	Standard Operating Procedure
SVR	Expert Council on Health Care

1 Rationale and Clinical Relevance

Demographic change with continuously increasing life expectancy is leading to a changing age structure, especially in Europe (Eatock et al. 2019; Fehr et al. 2018). A particular challenge is the provision of adequate nursing and medical care for the elderly and mostly multimorbid people in senior residences and nursing homes as well as in their personal home environment. The resulting increase in workload for nursing staff and doctors in the resident sector means that timely care for patients, e.g., in the form of GP visits, cannot always be guaranteed, especially in rural regions (Council of Experts 2018). The result is low-threshold acute admissions to hospitals as well as ambulance service and emergency doctor deployments, although in many cases it is not a matter of an acute or even life-threatening illness, but rather the gradual worsening of an already existing underlying condition through, for example, urinary tract infections, pneumonia or dehydration, so-called "ambulatory care sensitive hospitalizations" (ACSH) (Jacobs et al. 2018).

The disadvantages of this development are that the practitioner working in the hospital usually does not have the same knowledge of the medical history of the individual patient as the local doctor in private practice and, at the same time, the risk of confusion with resulting delirium increases for the patients due to the change of environment. Due to this development, among other things, the Expert Council on Health Care (Sachverständigenrat im Gesundheitswesen, SVR) recommended the necessity of a new regulation of intersectoral emergency care, including the outpatient and inpatient emergency service as well as the rescue service (Council of Experts 2018; German Medical Association 2018).

Optimal@NRW focuses on acute medical care of residents of care facilities. If a medical problem or a medical question currently arises in an inpatient care facility that exceeds the competences of the responsible nurse on site, the general practitioner is currently consulted by telephone and asked for advice. Outside the consultation hours of the GPs, the emergency medical service is available. This is often associated with long waiting times. If prompt care cannot be guaranteed, the ambulance service is usually called in to take the resident to an emergency room for further care, as on-site care is not possible in terms of time, personnel or logistics. The resident often remains in hospital afterwards. Out of 100 nursing home residents, about 20 persons are currently admitted to hospital once a year. Of these 20 retrospectively approx. 40 % are avoidable. They are referred to as ambulatory care sensitive hospitalizations (Jakobs et al. 2018; Sundmacher et al. 2015).

2 Aim of the trial

The aim is to improve the interlinking of the three sectors of outpatient care, inpatient care and emergency services. In this context, the medical acute and emergency care of patients in nursing homes in need of care is to be supported by the use of telemedical intersectoral network structures and optimised for care facilities in particular.

A core element is the merging of outpatient and inpatient emergency services in the form of portal practices. These are intended to create a central point of contact ("a one counter model") for the patient in order to ensure further care through needs-based triage. The project "Optimised acute care of geriatric patients using an intersectoral telemedical cooperation network - around the clock" (Optimal@NRW) starts even before these portal practices, in the sense of the "one counter model" for triage which is intended by the expert council.

The aim of the project is to improve medical care and avoid inadequate hospital admissions of nursing home residents through a graduated, intersectoral care network between geriatric nursing homes, the NRW 116117 medical call centre, resident doctors, hospitals and the doctor provided by Optimal@NRW at the Central Emergency Department at RWTH Aachen University Hospital and the emergency services.

The above-mentioned "one counter model" is thus supplemented by a central telemedical contact point ("virtual digital counter") for the patient and their caregivers. Through the bidirectional use of existing structures with the NRW 116117 medical call centre and the option of the tele-doctor of the Central Emergency Department at RWTH Aachen University Hospital, which work together as a care network, an early assessment of the nursing home residents takes place 24/7/365. The medical call centre uses the structured initial medical assessment (SmED) to ensure further care within the network according to the urgency.

If an emergency is detected, it is immediately forwarded to 112. For this purpose, the rescue coordination centres are connected to the 116117 medical call centre via a digital interface (see figure 1).

If there is no medical emergency, the NRW medical call centre forwards the patient primarily to the general practitioner or, if he or she is not available, to the medical on call service. If neither of these can be reached within 15 minutes, the telemedicine doctor from the Central Emergency Department at RWTH Aachen University Hospital is available as part of the innovative intersectoral telemedicine competence network. This doctor can carry out a teleconsultation and organise further assistance.

All participating physicians of the network can, if necessary, draw on the “non-medical practice assistance” provided by the network to support the staff of the nursing homes and to provide medically delegable services by visiting the residents on site.

For example, a dislocated bladder catheter, for which transport to a hospital was previously necessary or for which the general practitioner had to visit the nursing home, can be newly placed by using the non-medical .practice assistance.

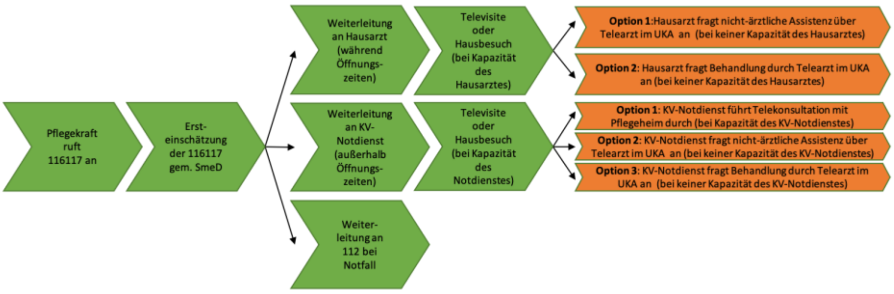

Figure 1: Overview of the supply process (German)

As can be seen in Figure 1, the project will not change the basic services. All processes that are already established are shown in green. The Optimal@NRW project provides options (see orange fields) when the capacities of the established care options are exhausted.

In addition, a standardised early warning system is to be established in the care facilities and its benefits evaluated. In this way, a potentially dangerous change in the state of health of nursing home residents can be recognised at an earlier stage. In order to avoid unnecessary hospital admissions and to improve medical care, standardised procedures will be developed with the partners nursing home, general practitioner, emergency medical service, hospital and ambulance service.

In summary, the Optimal@NRW innovation fund project aims to gain insights into which clinical pictures are suitable for this purpose and in which framework the project can contribute to a relevant medical improvement. The aim is to care for patients in local inpatient care in their familiar environment and, if possible, to leave them there, to achieve a corresponding reduction in ambulance missions, patient transports, emergency room treatments, inpatient treatments, hospital days, etc., while at the same time improving medical care and quality of life.

2.1 Primary target parameters

The primary outcome of the project is the number of hospital days, which includes all days that the trial participant spent in hospital within a defined period. This also includes stays with same-day discharge (especially cases of primary care in hospital with direct discharge). This is to ensure that all admissions are taken into account in order to reflect the primary goal of the intervention, the reduction of avoidable hospital stays. At the same time, the number of days that residents of care facilities can spend in their familiar surroundings should be increased (so-called "days spent at home").

2.2 Secondary target parameters

Secondary target parameters are:

- ICU length of stay
- Organ failure
- Mortality
- Hospital length of stay with regard to main diagnosis through telemedical visit after discharge
- Number of hospitalizations for ambulatory care-sensitive conditions (ACSH)
- 30-day survival/hospital survival
- nosocomial infections
- other

2.3 Further target parameters

In addition, a number of other outcomes are planned, which will be further specified in the synopsis. In addition to the duration of hospital stays, for example, the event "hospitalisation" (general + specified) will be used to support the primary outcome.

In addition, the patient transports used, subdivided into RTW, NEF/RTH and KTW, as a possible indication of a possible change in resource consumption as well as a possible change in the assessment of an emergency through the use of an upstream telemedical instance.

The use of the outpatient sector, which is also to be included in the intervention, is measured by the number of outpatient visits to the doctor and, additionally for the intervention group, the waiting time between contacting the outpatient doctor and the visit. By using the central electronic patient record as a communication platform, a coordinated process between nursing staff and doctors should also be achieved. This can have a positive effect on drug therapy safety.

These target parameters are supplemented by others.

2.4 Hypothesis

The provision and use of telemedicine equipment in care facilities and better intersectoral linkage can reduce hospital days for residents of care facilities while at the same time improving medical care.

2.5 Parallel evaluation strands

The project is assessed from parallel evaluation strands. In addition to the medical perspective, the project is evaluated from a health economic perspective. Furthermore, the evaluation of acceptance, ethics and usability is carried out. The latter are described in a separate protocol. The aim is to triangulate the medical-economic, ethical and user-relevant factors, in order to guarantee a holistic view. An overview and further information on the evaluation strands can be found in the following figure and the two additional test plans:

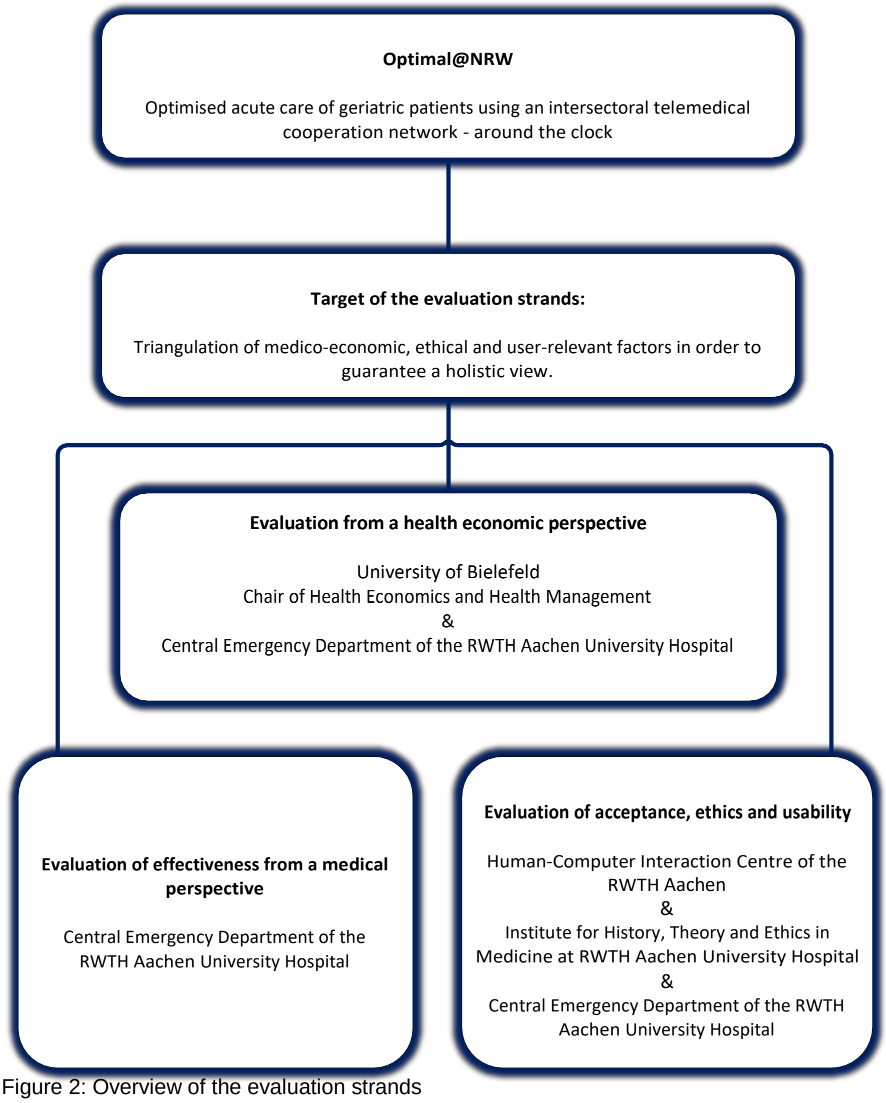

Figure 2: Overview of the evaluation strands

3 Trial design and duration

3.1 Trial design

This is a prospective, multicentre, cluster-randomised controlled intervention trial with a stepped-wedge design.

Month	1-3	4-6	7-9	10-12	13-15	16-18	19-21	22-24
Cluster 1								
Cluster 2								
Cluster 3								
Cluster 4								

Figure 3: Overview of stepped-wedge design; waiting period until intervention (white), intervention period (light orange), transition phase (grey)

The trial design is a cluster-randomised trial at the level of the nursing homes (inpatient sector) in the form of a stepped-wedge design. A consideration at the nursing home level supports the feasibility of the project, since a randomised allocation at the patient level would require the simultaneous implementation of the new form of care and the standard care within a facility (Brown & Lilford, 2006). This is not usefully realizable, because the internal validity of the trial results would be threatened by the Hawthorne effect (Gelman & Hill, 2006). In the stepped wedge design, observation of all clusters begins simultaneously, with all clusters initially in the control phase. Subsequently, the clusters move into the intervention phase at equal intervals, randomised in terms of timing. Thus, from an ethical perspective, the design offers the advantage that every participant receives the intervention until the end of the trial, as a positive added value of the project is assumed, while at the same time preserving randomised evidence through a randomised start of the intervention (Hemming, Haines, Chilton, Girling & Lilford, 2015). The stepwise establishment can also overcome logistical and practical implementation difficulties that arise in trial designs with simultaneous intervention start. Through a transition phase between the control and intervention phase, training measures for the nursing staff as well as technical installations can be carried out. The data collected during the transition phase are not included in the evaluation.

In the actual project, four clusters with a homogeneous number of nursing homes per cluster are formed, in order to ensure intra-cluster validity. In the sense of an 'Open Cohort', both longitudinal data (constant trial population over the entire course of the trial) and cross-sectional data (different patients in the individual stages) are included, in order to include the average mortality rates (Vossius, Selbæk, Šaltytė Benth & Bergh, 2018) of nursing home residents. The respective stages cover an interval of three months in terms of one quarter per year. In order to obtain a meaningful control and intervention group, the minimum number of surveys in both phases is two stages (six months).

In order to obtain a meaningful control and intervention group, the minimum number of survey in both phases is two stages (six months), so that the length of the control period of the first cluster corresponds to the length of the intervention period of the fourth cluster. The total anticipated duration of the control and intervention study is two years, resulting in a total of eight stages (see Fig 3).

3.2 Duration of trial

The trial will start with the education of the nursing home residents on 15.03.2021. The collection of data will start with the control phase on 01 May 2021 in all nursing homes. The first cluster enters the transition phase on 1 November and starts with the intervention phase on 1 February 2022. The following three clusters start with a time delay according to the scheme mentioned above. The end of the trial in all nursing homes is 30.04.2023.

4 Trial population

Residents (m/f/d) from the participating nursing homes (see nursing home list), who meet all inclusion criteria and no exclusion criteria, will be equally included in the trial.

4.1 Number of patients

N = approx. 2,184.

In total, more than 1700 residents live in the 24 care facilities participating in the Optimal@NRW project. With an expected dropout of approx. 12%, approx. 2.184 residents are ultimately included. Recruitment will take place permanently over the duration of the trial, if the residents drop out of the trial due to death or other reasons.

4.2 Inclusion criteria

1. Resident of one of the participating care facilities
2. Age of majority
3. Written declaration of consent
4. Consent of the guardian for residents who are not capable of giving consent

4.3 Exclusion criteria

1. Persons who are accommodated in an institution on the orders of the authorities or the courts.
2. Persons who are in a dependent or employment relationship with the investigator

5 Trial treatments and procedure

If a patient voluntarily consents in writing to participate in the trial and is eligible for participation according to all inclusion and exclusion criteria, the patient will be assigned a unique patient number that will be used to further identify all information collected on that patient from then on.

5.1 Project procedure

Virtual digital counter

Optimal@NRW initially relies on the already known and proven pillars of medical care (general practitioners, KV emergency service, rescue service), but expands them with a telemedical component. In a medical emergency situation, in which the immediate medical assistance by the emergency service does not have to take place, an upstream, intersectoral, telemedical instrument is used at the level of care facilities within the framework of Optimal@NRW.

The first step is to contact the emergency hotline of the Association of Statutory Health Insurance Physicians (116 117). The dispatcher first carries out a software-supported structured initial medical assessment using SmED (Structured Initial Medical Assessment in Germany).

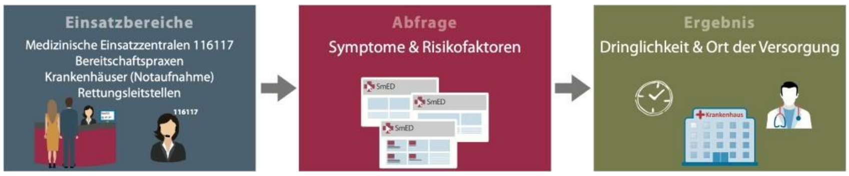

(Source: Central Institute for Statutory Health Insurance Physicians in Germany: https://www.zi.de/smed/)

Figure 4: Overview SmED workflow

The next steps in care provision within the cooperation network between the nursing facility, general practitioners, the NRW Physicians' Call Centre and the portal practice of the Association of Statutory Health Insurance Physicians of North Rhine-Westphalia (Kassenärztliche Vereinigung Nordrhein), which is currently being set up, the central emergency room of the RWTH Aachen University Hospital and the rescue services of the Aachen region are then examined, coordinated and initiated. The decisive factor is that the system builds on structures already established in standard care (see Figure 1 "green arrows") and supplements them with an intersectoral usable consultation and documentation system as well as the possibility of transferring measures that can be delegated by a doctor to a non-medical assistant (see below) (see Figure 1 "orange arrows").

Early warning system

As a supplementary component, a so-called early warning system (EWS) is implemented. With a voluntary daily and non-invasive measurement of certain vital values (e.g., blood pressure, pulse, oxygen saturation, temperature) on a so-called spot check monitor, the data obtained are compared within the EWS. In the event of an abnormality that indicates an imminent deterioration of the health condition, the EWS alerts the telemedicine unit of the RWTH Aachen University Hospital emergency room, in addition to the responsible nurse. In the best case, the developing medical problem is detected, before it becomes relevant and the resident is treated before he or she falls ill and, in the worst case, would have to be treated as an inpatient.

The EWS is already approved as a medical device and is only used within the scope of its approval.

Within the framework of the early warning system, the practicability of biosensors is to be investigated. Using a sensor system that is fixed to the skin (usually in the area below the collarbone) by means of a patch and left there for several days, vital parameters such as heart rate and rhythm, respiratory rate, oxygen saturation can be continuously detected and transmitted wirelessly to a monitoring station. Rare side effects to be expected from the systems known on the market consist of a potential intolerance to the patch, even though it is generally well tolerated. In order to exclude such intolerance, patients are asked about any known patch allergies. If a patch allergy is known, no biosensors are applied to this resident.

Non-medical assistance

If the need for a delegable activity becomes apparent during a teleconsultation (e.g., insertion of a transurethral bladder catheter), this activity can be performed by a non-medical assistant (m/f/d). The specially trained nurses are stationed at the Emergency Department of the RWTH Aachen University Hospital and are delegated to the respective nursing facility by car according to the doctor's orders (general practitioner, KV service, tele doctor of the RWTH Aachen University Hospital). For the Optimal@NRW project, training of the staff is specifically geared to the requirements of the project, in order to ensure a securely mastered implementation competence.

Teleconsultation system

Technically, a bidirectional communication structure is set up within the Optimal@NRW system, which enables data exchange in addition to communication in image and sound.
This teleconsultation system consists of a mobile stand, i.e., mobile into each resident's room, which is connected to the IT infrastructure via LAN/WLAN or mobile radio. The system is equipped with a monitor/input device combination for the nursing staff.

The doctor participating in the teleconsultation is displayed here by video. Furthermore, the patient's already stored data (electronic patient documentation and file) are displayed. The tele doctor's orders are transmitted in written form in conformity with the law. A conversation between the nurse and the tele doctor takes place via a monitor loudspeaker system.
The participating tele doctor is displayed to the patient via another monitor facing the resident. The tele doctor can also communicate with the patient via camera and loudspeaker/monitor. To record and display vital parameters in real time, the telecommunication system provides for the recording of blood pressure, peripheral oxygen saturation, ECG and temperature.

The Philips device used to record and transmit vital signs is approved as a medical device and is only used within the scope of its approval. This medical device is integrated into the overall software of the Docs in Clouds teleconsultation system.

Central electronic patient record

A central electronic patient record is also implemented, which can be viewed by all partners involved in the patient's treatment. Important medical information for the entire cooperation network is available there and can be integrated during treatment. Orders can be made in writing by the attending physician and their execution can be documented by the responsible nurse or non-medical assistant.

The medical decisions and measures taken as part of the teleconsultation, as well as the prescribed medication, are documented and stored in a central electronic patient record in accordance with the currently valid data protection regulations.

If the teleconsultation was carried out, for example, by the emergency physician of the emergency room at the University Hospital RWTH Aachen and not by the treating family doctor, the latter is informed accordingly by the system. This means that he or she not only has the possibility to inspect and check the measures carried out, but can also coordinate the further therapy steps and, if necessary, follow and shape the medical course by means of another teleconsultation.

6 Data collection

The following data will be collected at the beginning of the trial:

- Written consent
- General information about the resident (health insurance, legal care, name of the nursing home, family doctor, power of attorney/ living will, etc.)
- Demographic data (name, age, gender, ethnicity, etc.)
- Medical information about the resident (previous illnesses, allergies, home medication, ECG, vaccinations, etc.)
- Size, weight

- Survey of doctor contacts and hospital admissions retrospectively since the start of the study (at the earliest from 01.05.2021).
- Quality of life surveys
- more

The following data are collected during the control phase/transition phase/intervention phase:

- Hospitalisation (number of hospital days, admission diagnosis, etc.)
- Visit to family doctor, specialist, etc. (date, duration, occasion, etc.)
- Rescue service mission (date, occasion, etc.)
- more

The following data will be collected during the intervention phase:

- Early Warning System data
    - Blood pressure
    - Heart rate
    - Oxygen saturation
    - Temperature
    - Respiratory rate (when using biosensors)
    - more
- Tele visit:
    - Reason for the tele visit
    - Time of request, start and end of the tele visit
    - Output of the tele visit
        - Use of the non-medical assistant
    - Content of the tele visit
        - Survey according to medical practice and symptoms
            - Vital data
            - Vigilance
            - Fast Test
            - Pupil status
            - Auscultation
            - Indication of delirium
            - 12-channel ECG
    - more

The following data is collected in the event of a drop-out:

- Reason for leaving
    - Death
    - Transfer to another nursing home, if this is not part of the Optimal@NRW project.
    - Withdrawal of the declaration of consent
    - more

These data will be collected in a trial database by the nursing staff as well as by the other medical service providers participating in the project. These data will be supplemented by routine data from the participating health insurance funds from 2019, which they will make available for the participating insured persons. The preliminary observation period of two years is intended to determine the relevant baseline characteristics of the patients.

The data from the central electronic patient record are transferred to a study database. By means of a trusted third party, the primary data are pseudonymised and another pseudonym key is made available to the health insurance funds. The merging of the data sets takes place at the evaluation centre in pseudoymised form.

7 Safety data, records and notifications

Patient safety is monitored and assessed through regular monitoring and a Safety Board.

7.1 Safety Board

The Safety Board decides whether the safety of the patients is still guaranteed. The Safety Board consists of independent doctors who are not involved in the implementation of the project. The Safety Board is alerted in case of the following events:

- Resuscitation during teleconsultation
- Unexpected death during teleconsultation
- Unexpected death within 24 hours of a teleconsultation
- Unexpected hospitalisation within 24 hours of a tele-consultation
- Unexpected death while wearing a biosensor (if available)

7.2 Documentation of events that could potentially jeopardise patient safety

Adverse events or serious adverse events are documented, evaluated and reported in accordance with GCP and the Professional Code of Conduct for Physicians (BOÄ).

8 Termination of trial

The trial participation of an individual trial participant can be terminated prematurely. if one of the following criteria is fulfilled:

- Serious adverse events that do not allow the continuation of the trial
- Lack of compliance
- The trial participant withdraws the declaration of consent

The reason, timing and specific details of a participant's withdrawal from the trial are recorded on a separate page of the CRF (comments page). The investigator should specify a primary reason for the participant's withdrawal from the trial.

All safety-relevant data are collected and reported until the trial participant withdraws from the trial.

Any decision regarding early trial termination for a trial participant or his/her exclusion will be discussed in advance with the clinical trial director. Dropout trial participants will be replaced.

The entire trial may be discontinued if one of the following criteria is met:

- The benefit-risk assessment on which the trial is based changes in such a way that the risks outweigh the benefits and/or the potential risks are no longer justifiable.
- Administrative problems that prevent the proper conduct of the trial.
- Serious protocol violations

9 Further treatment plan

No treatments will be given after the end of the trial. All trial participants return to their usual standard patient care after completion of the trial. This also applies to participants who have withdrawn their consent during the ongoing trial.

10 Statistics

10.1 Case number calculation and sample size

The average number of hospital days of persons in need of care is 13.16 days per quarter (Jacobs, Kuhlmey & Greß, 2020). Taking into account the age structure >70 years (Federal Statistical Office, 2019), this results in an underlying average number of hospital days of 13.22 for the defined trial population. For the case number calculation, it is assumed that this can be reduced by 30% to 9.25 days. The assumption of the effect size was quantified by the specialists of the University Hospital Aachen. The internal dependence of the individual clusters is estimated with an intra-class correlation coefficient (ICC) of $\rho=0.12$, which is based on the ICC calculation of a preliminary trial by Hoffmann & Schmiemann (2017) for cluster-based studies in nursing home settings as well as the consideration of the trial design with several nursing homes in one cluster. With an α-error of 0.05, a power of $1-\beta = 0.90$ and an assumed standard deviation of 4.5, an underlying linear regression model results in a case number of 43 nursing home residents per nursing home and time interval. Due to the high mortality rate of the trial population (Vossius et al., 2018) and further factors of a possible dropout, a quarterly dropout of 12% is assumed, so that the number of cases increases to 49 nursing home residents per nursing home and time interval. This corresponds to a quarterly additional recruitment of approx. 6 nursing home residents per nursing home. This results in a total number of cases of **N=2,184 persons**. Expressed differently, this means a number of cases of n=1,173 per cluster, which means that N=10,250 observations are available for all persons across all clusters, which result from both longitudinal data of n=1,032 persons and the n=1,152 persons additionally recruited as cross-sectional data. Distributed over the individual phases of the trial design, this results in a

number of cases at observation level of n=4,105 in the control group, n=1,173 in the transition phase and n=4,105 in the intervention phase, so that at person level the above-mentioned number of cases of 49 nursing home residents per nursing home and time interval results. To ensure the internal validity of a cluster, it must be ensured that the number of nursing homes and their residents are almost homogeneously distributed.

11 Ethical and legal aspects

11.1 Independent Ethics Committee

The implementation of the present project is subject to the Professional Code of Conduct for Physicians (BOÄ).

The project will be implemented according to the ethical principles originating in the Declaration of Helsinki.

The head of the clinical trial submits an application for a favourable evaluation to the ethics committee. The clinical trial may only be started, if the responsible ethics committees have given their approval.

Any changes to the protocol and / or the trial participant information and consent form will be resubmitted to the relevant ethics committees for consideration. They must be approved by the Ethics Committee before implementation (exception: logistical or administrative changes or to avoid direct hazards).

The investigator or the head of the clinical trial receives a copy of the positive evaluation (ethics vote).

11.2 Information and declaration of consent

According to the GCP guidelines informed consent must be obtained from the patient, before participation in the trial.

Patients voluntarily confirm their willingness to participate in the trial after they have been informed verbally and in writing by a physician about all aspects of the trial that are relevant to the decision to participate. They are informed about data protection regulations and must agree to direct access to their personal data.

The trial participants sign a trial participant information and consent form for participation in the trial and disclosure of personal data. The trial participant information and consent form must be personally signed and dated by the trial participant and a clinical trial doctor.

Before obtaining a declaration of consent, the clinical trial doctor must allow the trial participant sufficient time and opportunity to ask questions about details of the trial and to decide whether or not to participate in the trial. All questions about the trial must be answered to the trial participant's satisfaction.

The information provided to the subject/patient must include at least the following points:

- Procedure and objectives of the trial
- Planned duration of participation

- Expected benefit
- Possible risks or inconveniences
- Alternative methods of treatment
- Responsibilities of the trial participant
- Voluntariness of participation
- Withdrawal of consent at any time and without adverse consequences
- Collection and use of personal data
- Disclosure in pseudonymised form to authorities
- Publication of selected data in anonymised form (Open Data)
- Inspection of personal medical data by authorized representatives of the principal or the authorities
- Approximate total number of trial participants

The trial participants will receive a copy of the signed consent form.

The trial participants will be informed by an clinical trial doctor in a timely manner, if new information becomes known that could be relevant to their willingness to continue participation in the trial. The disclosure of this information is documented. The patient will receive a copy of any changes or additions to the trial participant information and a copy of the newly signed and dated informed consent form.

Trial participants must be informed that they are free to withdraw their consent to participate in the trial, without any adverse consequences, at any time and without giving reasons..

There will be joint information and declaration of consent for the three evaluation strands.

11.2.1 Patient ID card and care record labelling

Each trial participant will receive a patient ID card and will be required to carry it with them at all times. The patient ID card contains the following information:

- Name, address and telephone number of the investigator/facility

In addition, it is marked in the resident's care file that they are participating in the Optimal@ NRW project.

11.3 Delegation of auditor duties

The clinical trial doctor should ensure that all persons assisting him/her in the trial are adequately qualified and informed about the protocol, any amendments to the protocol, the intervention methodology and their trial-related duties and functions.

The clinical trial doctor shall maintain a list (Trial Staff Authorisation Log) of all persons to whom they have delegated trial-related duties.

11.4 Test plan changes

Amendments to a clinical trial that has been assessed with approval by the competent ethics committee and which are able to

- have an impact on the safety of the persons concerned,
- influence the interpretation of the scientific documents on which the trial is based or the scientific validity of the trial results,
- substantially change the way the trial is managed or conducted,

may only be made, if these changes have been approved by the competent ethics committee.

Any changes to the protocol will be signed by the clinical trial director (LKP).

11.5 Data protection

It is ensured that patient-related data will not be disclosed to third parties at any time. The evaluation of trial results is carried out exclusively with pseudonymised or anonymised data. Publication of the data is anonymous. The relevant data protection regulations in accordance with the Federal Data Protection Act are taken into account and ensured at all times. For open data publications, selected or processed original data is published anonymously.

The data stored in the central electronic patient file will be made available to the participating scientists of the Optimal@NRW Research Group for answering the research questions in this project.

The data is only transmitted to the research institutes in pseudonymised form and stored there.

The consent and thus information about the patient (surname, first name, date of birth, health insurance company and insurance number, as well as contact details) are transmitted to a trusted third party and stored there with the pseudonym for the data. At no time does the trusted third party gain an insight into your medical data or the health insurance data.

The health insurance data are requested by the trusted third party with the help of the insurance number. The health insurance companies then make their medical data available in such an encrypted form that it can only be viewed by the evaluation centre. However, the evaluation centre only receives the data with a pseudonym and thus cannot infer the person. Only employees of the evaluation centre have access to the total data set, which is not passed on.

After completion of the trial, proper storage and administration of all trial documents will be ensured for at least ten years in accordance with the statutory provisions. Deletion will take place no later than 31.12.2036. After this date, personal data will be deleted, unless legal, statutory or contractual retention periods conflict with this.

The proper implementation of the project, in particular the proper collection of the data and their allocation to specific probands, is also checked by at least one authorised representative of the client, also by direct inspection of the non-pseudonymised data available at the investigator's office in the presence of the contact person at the trial centre. The authorised representative of the project client will be

specially trained for this task. They have undertaken in a written declaration to maintain confidentiality - also towards the client of the project. The authorised representative also observes the applicable data protection laws. They may only pass on data/illness data in pseudonymised form and may not make copies or transcripts of the illness documents.

The competent supervisory authority, other authorities or the ethics committee may also have access to the personal data held by the trial centre. Personal data will be treated confidentially in accordance with the applicable data protection law.

The consent form contains the patient's name in print and signature and is also kept separately from the patient's medical data in the trusted third party in a lockable cabinet.

The authorised group of persons who have access to the personal data may not make copies of the patient identification list or the signed patient consent forms at any time.

11.5.1 Collection, access and use of personal data

The participants are informed about how data protection is ensured. The data recorded will only be passed on by the investigator to authorised persons in encrypted or anonymised form.

Participants have the right to inspect their data collected during the trial. If errors are discovered in their data, participants have the right to have these corrected by the clinical trial doctor. Furthermore, participants have the right to information and to be provided with a copy of their data. Participants also have the right to complain to a supervisory authority about the handling of their data.

Access to unencrypted data is only granted in order to check the validity of the data. This is only possible for strictly limited persons (authorised persons, auditors, members of the ethics committee, competent supervisory authorities) who are committed to confidentiality.

All participants can be identified via a unique pseudonymisation number. The trusted third party has lists for the identification of the trial participants. These lists are kept secure and protected from unauthorised access by third parties. In addition, the declaration of consent signed by the trial participants is stored in the trusted third party.

Personal data, in particular health data, may, to the extent required

- be kept available for inspection by the supervisory authority to verify the proper conduct of the clinical trial,
- be passed on to a commissioned body for the purpose of scientific evaluation in pseudonymised form,
- be passed on to the trusted third party to merge the medical data and the health insurance data.

12 Quality assurance and control

In order to ensure the use of standardised terminologies and procedures, as well as the collection of accurate, consistent and trustworthy data, this project will be planned and conducted according to the SOPs of the CTC-As quality management system. The CTC-As quality management system, together with the IZDM project team, implements all requirements for planning, conducting and completing a GCP-compliant research project, taking into account all ethical and legal provisions and high-quality objectives, to ensure the well-being of project participants and the collection of valid, objective and reliable data.

12.1 Documentation of original data

All collected data are listed in the central electronic patient file. A copy of the letter to the family doctor about the trial participation should also be filed there, if the notification of the family doctor was requested by the trial participant.

Trial participation must be adequately documented (trial number, trial participant number, date of informed consent, date of consent).

The trial database will not be closed after the end of the trial until the collected data or the data management has been quality assured.

12.2 Data management

All data to be collected (e.g., medical history, existing therapies, blood pressure measurements, pulse measurements, ECGs, etc.) are entered into the central electronic patient record and transferred to the eCRF. If it is necessary, to collect data on raw data lists, the data are transferred to the eCRF and the raw data lists are regarded as source data. Data that are created automatically and are available as original printouts are transferred manually to the eCRF.

12.3 Direct access to the original data

The investigator is obliged to allow trial-specific monitoring and to support it to the best of their ability. They must ensure direct access to all original data and source documents.

12.4 Monitoring

This trial is regularly reviewed by a qualified monitor of the CTC-A in accordance with GCP. This also includes one or more visits before the start of the trial, in order to check all prerequisites and, if necessary, to initiate appropriate measures. The monitoring is intended to detect and eliminate existing deficiencies. The auditors and all staff members support the monitor and, if necessary, provide all necessary information relevant to the performance of the monitoring or ensure access to the original data. During the monitoring visit, an auditor is available to answer questions.

As part of the monitor visits during the ongoing trial, the monitor will review the following processes:

- Entries of the data in the eCRFs

- Protocol compliant processes
- Compliance with ICH-GCP guidelines, Declaration of Helsinki and official legal requirements
- Integrity of the original data with the entries in the eCRF
- Signed consent forms
- Correct documentation or reporting of AEs and SAEs
- Maintained trial lists (e.g., screening/enrolment log)

Further details on monitoring are continued in the Monitoring Manual.

13 Handling, documentation and archiving of original data

13.1 Documentation completion

By signing the eCRF/eSignature, the investigator confirms that the contents of the clinical trial have been completed and carried out in accordance with the trial protocol and that all data have been entered correctly and completely.

13.2 Data correction

If a correction of paper-based data is necessary, this is done by crossing out the original value with the help of a line. The correct value is written next to it, with the date and name abbreviation as stored in the investigator's folder.

The clinical trial doctor is responsible for data processing according to the procedures implemented for data management. The database (electronic Case Report Form) will only be closed after all quality assurance procedures have been completed.

13.3 Archiving of trial documents

The investigator will retain all trial-specific data for as long as possible and in accordance with local methods and premises. The investigator should retain the trial documents specified in the ICH GCP guidelines (Section 8, Essential Documents) for at least 2 years. Precautions are taken to prevent accidental or premature destruction of these documents.

Trial-specific documents, patient identification lists and original consent forms are archived for at least 10 years after the end of the trial in accordance with the GCP regulation.

The essential documents relevant for archiving include:

- Patient files
- Patient identification list
- Original signed protocol and all amendments
- CD/DVD with eCRF data or patient-related source data
- Original consent forms
- Curricula vitae of all registered investigators
- Documents for correspondence with ethics committees and competent authorities
- Correspondence between monitor and investigator

13.4 Destruction of trial documents

Trial documents will not be destroyed before the archiving period has expired. Following this period, trial documents may only be destroyed with the written consent of the head of the clinical examination.

14 Publication

The trial will be registered at ClinTrial.gov and the trial results will be published in one or more scientific journals.

The Ethics Committee is informed of the trial results.

15 Financing and insurance

15.1 Funding

The project is funded by the Innovation Fund of the Federal Joint Committee for the Promotion of New Forms of Health Care (§92a para. 1 SGB V). The funding period of the full funding starts on 01 April 2020 and ends on 31 March 2024.

Funding code: 01NVF19015

The details can be found in the funding decision.

15.2 Insurance

No additional patient insurance has been taken out. In case of incidents due to negligence, the patients are covered by the usual liability insurance of the hospital.

The University Hospital of RWTH Aachen has a public liability insurance policy with Allianz Versicherungs-AG (insurance number: AS-9104141415).

A road accident insurance policy has not been taken out.

16 Declaration of conformity

I have read the observation plan in detail and checked it for accuracy and completeness. I have understood the requirements and conditions of this research project and assure that I will carry out the project in accordance with this observation plan, the Helsinki Declaration and the applicable legal provisions (§ 15 BOÄ, BDSG).

I also agree,

- to wait for a favourable assessment by the relevant ethics committee, before including participants in this project.
- to carry out project-related actions only after obtaining the written consent of the participant.
- to submit any change to the observation plan as an amendment to the competent ethics committee for a favourable evaluation.

- to allow project-related monitoring and audits by an authorised representative of the CTC- A and ensure access to all original data, records and medical records at all times.
- to treat project contents confidentially.

17. Signatures

The audit plan is approved:

Principal Investigator

**PD Dr. med. Jörg Christian Brokmann**	Aachen, the 5th of January 2022
Senior physician of the Central Emergency Department	
Uniklinik RWTH Aachen	

18. Literature

Brown, C. A. & Lilford, R. J. (2006). The stepped wedge trial design: a systematic review. *BMC medical research methodology, 6,* 54.

Bundesärztekammer. Beschlussprotokoll des 121. Deutschen Ärztetages in Erfurt vom 08. bis 11.05.2018, Stand 08.06.2018.

Eatock D. Demografischer Ausblick für die Europäische Union 2019.

Fehr A, Lange C, Fuchs J, Neuhauser H, Schmitz R. Gesundheitsmonitoring und Gesundheitsindikatoren in Europa. Robert Koch-Institut, Epidemiologie und Gesundheitsberichterstattung; 2017.

Gelman, A. & Hill, J. (2006). *Data Analysis Using Regression and Multilevel/Hierarchical Models*. Cambridge: Cambridge University Press.

Hemming, K., Haines, T. P., Chilton, P. J., Girling, A. J. & Lilford, R. J. (2015). The stepped wedge cluster randomised trial: rationale, design, analysis, and reporting. *BMJ (Clinical research ed.), 350,* h391.

Hoffmann, F. & Schmiemann, G. (2017). Influence of age and sex on hospitalization of nursing home residents: A cross-sectional study from Germany. *BMC health services research, 17* (1), 55.

Jacobs K, Kuhlmey A, Greß S, Klauber J, Schwinger A. Pflege-Report 2018. Berlin, Heidelberg: Springer Berlin Heidelberg; 2018.

Sachverständigenrat zur Begutachtung der Entwicklung im Gesundheitswesen. Bedarfsgerechte Steuerung der Gesundheitsversorgung. Gutachten 2018

Statistisches Bundesamt. (2019). *Statistisches Jahrbuch 2019:* Statistisches Bundesamt.

Sundmacher, L., Fischbach, D., Schuettig, W., Naumann, C., Augustin, U. & Faisst, C. (2015). Which hospitalisations are ambulatory care-sensitive, to what degree, and how could the rates be reduced? Results of a group consensus study in Germany. *Health policy (Amsterdam, Netherlands), 119* (11), 1415-1423.

Vossius, C., Selbæk, G., Šaltytė Benth, J. & Bergh, S. (2018). Mortality in nursing home residents: A longitudinal study over three years. *PloS one, 13* (9), e0203480.

Formatiert: Einzug: Links: 0 cm
